# The displacement of the σ^70^ finger in initial transcription is highly heterogeneous and promoter-dependent

**DOI:** 10.1093/nar/gkaf857

**Published:** 2025-09-12

**Authors:** Anna Wang, Andrew Fletcher, Pratip Mukherjee, David C Grainger, Abhishek Mazumder, Achillefs N Kapanidis

**Affiliations:** Biological Physics Research Group, Department of Physics, University of Oxford, Oxford OX1 3PU, United Kingdom; Kavli Institute for Nanoscience Discovery, Dorothy Crowfoot Hodgkin Building, University of Oxford, Sherrington Road, Oxford OX1 3QU, United Kingdom; School of Biosciences, University of Birmingham, Edgbaston, Birmingham B15 2TT, United Kingdom; Structural Biology and Bioinformatics Division, CSIR-Indian Institute of Chemical Biology, 4 Raja S. C. Mullick Road, Jadavpur, Kolkata-700032, India; Academy of Scientific and Innovative Research (AcSIR), CSIR-Human Resource Development Centre, Ghaziabad 201002, India; School of Biosciences, University of Birmingham, Edgbaston, Birmingham B15 2TT, United Kingdom; Biological Physics Research Group, Department of Physics, University of Oxford, Oxford OX1 3PU, United Kingdom; Kavli Institute for Nanoscience Discovery, Dorothy Crowfoot Hodgkin Building, University of Oxford, Sherrington Road, Oxford OX1 3QU, United Kingdom; Structural Biology and Bioinformatics Division, CSIR-Indian Institute of Chemical Biology, 4 Raja S. C. Mullick Road, Jadavpur, Kolkata-700032, India; Biological Physics Research Group, Department of Physics, University of Oxford, Oxford OX1 3PU, United Kingdom; Kavli Institute for Nanoscience Discovery, Dorothy Crowfoot Hodgkin Building, University of Oxford, Sherrington Road, Oxford OX1 3QU, United Kingdom

## Abstract

Most bacterial sigma factors (σ) contain a highly conserved structural module, the ‘σ-finger’, which forms a loop that protrudes towards the RNA polymerase active centre in the open complex and has been implicated in pre-organization of template DNA, abortive initiation of short RNAs, initiation pausing, and promoter escape. Here, we introduce a novel single-molecule FRET (smFRET) assay to monitor σ-finger motions during transcription initiation and promoter escape. By performing real-time smFRET measurements, we determine that for all promoters studied, displacement occurs before promoter escape and can occur either before or after a clash with the extending RNA. We show that the kinetics of σ-finger displacement are highly dependent on the promoter, with implications for transcription kinetics and regulation. Analogous mechanisms may operate in the similar modules present across all kingdoms of life.

## Graphical Abstract

**Graphical Abstract:**
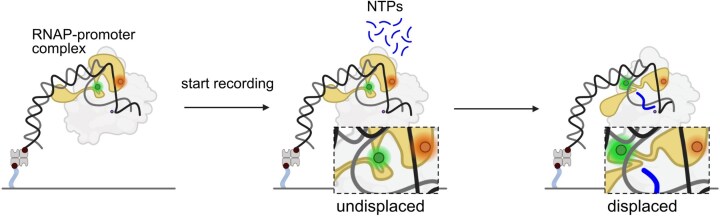

## Introduction

All cellular core RNA polymerases (RNAP) require at least one protein factor to perform promoter-specific transcription initiation [1–3]. In bacterial transcription, the core RNAP associates with a σ-factor (either the primary σ [σ^70^] or alternative σ-factors [such as σ^24^, σ^28^, σ^32^, σ^38^, and σ^54^]) to carry out promoter-specific transcription initiation [1]. Nearly all RNAP-σ holoenzymes (except RNAP-σ^54^; see below) contain a structurally similar module called the ‘σ-finger’ (also referred to as ‘σR3-σR4 linker’ or ‘σR2-σR4 linker’), which is positioned inside the active centre cleft of RNAP and interacts with unwound template strand DNA [4–8]. The RNAP-σ^54^ contains a structurally unrelated module, RII.3, which occupies a position analogous to that of the σ-finger module, and has been hypothesized to play a similar functional role [9]. Remarkably, all three domains of life feature σ-finger-like structural elements that protrude into the RNAP active-centre cleft to interact and stabilize the unwound template-strand DNA in conformations that facilitate the binding of initiating nucleotides [2–17].

**Figure 1.**
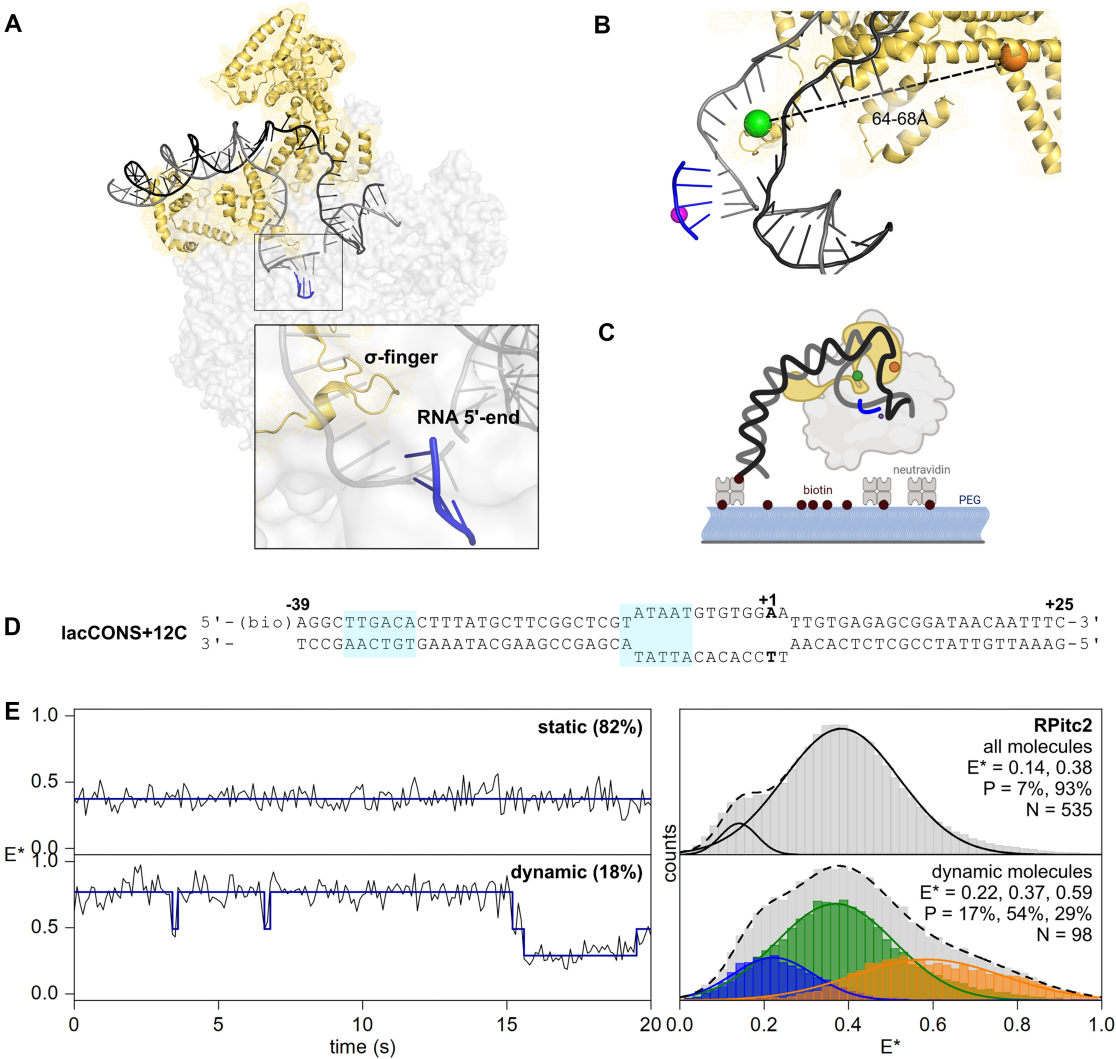
An smFRET assay for detecting σ-finger movements in solution. (**A**) Conformational state of the pre-displaced σ-finger in *Escherichia coli* RP_itc4_ (PDB 4YLN). Grey, black, and blue ribbons show the template DNA strand, non-template DNA strand, and transcribed RNA, respectively. Straw ribbons and mesh show σ^70^. Created in BioRender. Mazumder, A. (2025) https://BioRender.com/22tuag8. (**B**) smFRET labelling scheme. Orange and green spheres: fluorescent probes at residues 511 and 366 of σ^70^. Purple sphere: catalytic Mg^2+^ ion. Created in BioRender. Mazumder, A. (2025) https://BioRender.com/22tuag8. (**C**) Schematic of the assay used for experiments tracking σ-finger movements. Colour scheme as in panel (A) but with the addition of RNAP core shown in light grey. (**D**) lacCONS DNA fragment used for the assay to track σ-finger movements at RP_itc2_. (**E**) smFRET data for the σ-finger in RP_itc≤2_ complexes formed using ApA dinucleotide primer showing static (upper) and dynamic (lower) behaviour. Left, representative traces of static and dynamic behaviour. Right, E* histograms are formed as a result of hidden Markov modelling and Gaussian fitting of sub-populations.

Observation of high-resolution structures of bacterial transcription initiation complexes showed that the σ-finger is found along the path of the growing RNA chain (Fig. 1A); it has thus been postulated for a long time that the σ-finger needs to be displaced from the path of the RNA to allow for productive transcription, with the displacement thought to occur either before or during RNA synthesis in initial transcription [4, 18–20]. Consistent with the structural studies, biochemical studies have reported altered transcription profiles for RNAP-σ holoenzymes containing σ-finger mutations or deletions [4, 21, 22]; further, a recent single-molecule study on the kinetics of initial transcription reported significant changes in pausing profiles during initial transcription by an RNAP derivative with a σ-finger deletion [23].

Further insight on σ-finger interactions and conformations during initiation was provided by a recent study that reported several high-resolution structures of *Thermus thermophilus* RNAP and *Mycobacterium tuberculosis* RNAP transcription initiation complexes and defined σ-finger interactions with a DNA–RNA hybrid containing different lengths of RNA [18]. The reported structures indicated stabilization of very short DNA–RNA hybrids, also reported in [24], and stepwise displacement of the tip of the σ-finger from the initial position in the active site cleft as the RNA length increases (>4 nt) and the RNA clashes with the σ-finger. Based on these structures, it has been hypothesized that the σ-finger acts as a ‘protein spring’ by folding back on itself, storing energy that is utilized later during the large-scale disruption of RNAP-promoter and RNAP-σ interactions that occurs in promoter escape. However, the study used nucleic acid scaffolds that did not contain a scrunched DNA [25] as would be found in actual initial transcribing complexes. As a result, the exact mechanism and kinetics of σ-finger displacement during active transcription in the context of a full promoter remain unresolved.

In this work, we define the σ-finger conformation and dynamics at different stages of transcription initiation—the open complex (RP_o_), initial transcribing complexes (RP_itc_), and an elongation complex (RD_e_)—for three well-characterized promoters with different properties: a consensus bacterial promoter based on lac promoter (lacCONS), a phage λ promoter (pR), and a ribosomal RNA promoter (rrnBP1). While lacCONS is a promoter with a consensus −10 element, a consensus −35 element, and a consensus 17 bp spacer (Fig. 1D); pR and rrnBP1 are naturally occurring promoters with characteristically different properties. The pR promoter is characterized by a consensus 17-bp spacer and an AT-rich downstream promoter sequence with promoter escape occurring relatively late after synthesis of ∼12–13 nt long RNA [23]; while rrnBP1 is characterized by a shorter (16-bp) spacer and GC-rich downstream promoter segment with promoter escape occurring earlier after synthesis of ∼3–4 nt long RNA [26]. Using single-molecule FRET (smFRET) approaches, we identify a new ‘displaced’ σ-finger conformation, define the point of σ-finger displacement for different promoters, identify determinants of σ-finger displacement, and measure the kinetics of σ-finger displacement during active transcription. Our results show surprising diversity between promoters regarding the RNA length at which σ-finger displacement occurs and reveal the presence of RP_o_ molecules with an order of magnitude difference in σ-finger displacement kinetics during initial transcription.

## Materials and methods

### Accessible volume calculations

Accessible volume calculations for DL RNAP-σ^70^ (labelled at σ^70^ residue 366 Ser and residue 511 Ile) were carried out on crystal structures PDB 4G7H, 6KQD, 6KQE, 6KQF, 6KQG, 6KQH, 4YLN, 7KHB, 7MKD, 7MKE, and 7KHC using FPS software [27]. Amino acid side chains on σ^70^ residues 511 and 366 (4YLN, 7KHB, 7MKD, 7MKE, and 7KHC), on σ^A^ residues 321 and 174 (6KQD, 6KQE, and 6KQF), and on σ^A^ residues 319 and 174 (4G7H, 6KQD, 6KQE, 6KQF, 6KQG, and 6KQH) were deleted in all structures. The dye attachment point used was Cα. Dye parameters for accessible volume measurements were as follows: Alexa647 maleimide (linker length = 21 Å, linker width = 4.5 Å, and dye radii = 11.0 Å, 4.7 Å, and 1.5 Å) and Cy3B maleimide (linker length = 9.1 Å, linker width = 4.5 Å, and dye radii = 7.7 Å, 2.5 Å, and 1.3 Å). Average distances and corresponding FRET values were then calculated using a Förster radius of 60 Å.

### Preparation of reagents

#### σ^70^ derivatives

Double cysteine-modified σ^70^ derivatives were prepared as follows: Single colonies of *E. coli* strain BL21(DE3) (Millipore) were co-transformed with a pGEMD (-Cys) derivative encoding two cysteine residues at positions 366 and 511 [constructed from plasmid pGEMD (-Cys) [28], which does not encode for any cysteine residue by use of site-directed mutagenesis (QuikChange Site-Directed Mutagenesis Kit; Agilent) to replace codons 366 and 511 by a codon encoding cysteine residue], were used to inoculate 20 ml LB broth containing 100 μg/ml ampicillin, and cultures were incubated 16 h at 37°C with shaking. Culture aliquots (2 × 10 ml) were used to inoculate LB broth (2 × 1 L) containing 100 μg/ml ampicillin; cultures were incubated at 37°C with shaking until OD_600_ = 0.7; IPTG was added to 1 mM; and cultures were further incubated for 4 h at 37°C with shaking. Cells were harvested by centrifugation (5000 x *g*; 20 min at 4°C), resuspended in 50 ml lysis buffer [40 mM Tris–HCl (pH 7.9), 300 mM NaCl, 1 mM ethylenediamine tetraacetic acid (EDTA), one protease inhibitor cocktail tablet, and 0.2% deoxycholate], and lysed by emulsification (Emulsiflex-C5; Avestin, Inc., Ottawa, Canada). Inclusion bodies containing σ^70^ derivatives were isolated by centrifugation (10 000 × *g*; 20 min at 4°C), washed with 20 ml lysis buffer containing 0.2 mg/ml lysozyme and 0.5% Triton X-100, and washed with 20 ml lysis buffer containing 0.5% Triton X-100 and 1 mM DTT [with each wash step involving sonication 2 × 1 min at 4° in wash buffer, incubation 10 min at 4°C in wash buffer, and centrifugation (10 000 × *g*; 20 min at 4°)]. Washed inclusion bodies containing σ^70^ derivatives were solubilized in 40 ml 6 M guanidine–HCl, 50 mM Tris–HCl (pH 7.9), 10 mM MgCl_2_, 10 μM ZnCl_2_, 1 mM EDTA, 10 mM DTT, and 10% glycerol and dialyzed against 2 L TGED [20 mM Tris–HCl (pH 7.9), 0.1 mM EDTA, 0.1 mM DTT, and 5% glycerol] containing 0.2 M NaCl (20 h at 4°; two changes of buffer). The sample was centrifuged (10 000 × *g*; 20 min at 4°) to remove particulates and applied to a Mono-Q HR 10/10 column (Amersham-Pharmacia Biotech, Piscataway, NJ) pre-equilibrated in the same buffer. The column was washed with 16 ml of the pre-equilibration buffer and eluted in 2-ml fractions of a 160-ml linear gradient of 200–600 mM NaCl in TGED (with σ^70^ derivatives typically eluting at ∼360 mM NaCl in TGED). Fractions containing the σ^70^ derivative were identified by sodium dodecyl sulphate–polyacrylamide gel electrophoresis and Coomassie staining and are pooled. Pooled fractions were concentrated in storage buffer [20 mM Tris–HCl (pH 7.9), 200 mM NaCl, 0.1 mM EDTA, 1 mM TCEP, and 5% glycerol] using 10 kDa MWCO Amicon Ultra-15 centrifugal ultrafilters and stored in aliquots at −80°C.

Fluorescent-probe-labelled σ^70^ derivatives were prepared as follows: A reaction mixture containing 10 μM σ^70^ derivative [with cysteines at positions 366 and 511], 500 μM Cy3B maleimide, and 500 μM Alexa647 maleimide in 0.5 ml buffer B (100 mM potassium phosphate buffer pH 8.0, 50 mM NaCl, 1 mM EDTA, 5 mM TCEP, and 2% dimethylformamide) was incubated for 4 h on ice, subjected to 5 cycles of buffer exchange dilution with 5 ml buffer B (without dimethylformamide), followed by concentration to 0.5 ml using 10 kDa MWCO Amicon Ultra-15 centrifugal ultrafilters (EMD Millipore), and stored in aliquots at −80°C.

Efficiencies of incorporation of fluorescent probes were determined from UV/Vis-absorbance measurements and were calculated as:


\begin{eqnarray*}
{\rm concentration}\;{\mathrm{ }} {\rm of}\;{\mathrm{ }}{\rm product} &=& \left[{A_{280}} - {\varepsilon_{Cy3B,280}}({A_{Cy3B,570}}/{\varepsilon_{Cy3B,570}}){\mathrm{ }} \right.\nonumber\\ &&\left.- {\varepsilon_{Alexa647,280}}({A_{Alexa647,665}}/{\varepsilon_{Alexa6470,665}})\right]/{\varepsilon_{P,280}} ,
\end{eqnarray*}

\begin{eqnarray*}
{\rm Cy3B}\;{\mathrm{ }}{\rm labelling}\;{\mathrm{ }}{\rm efficiency}\;{\mathrm{ }} = {\mathrm{ }}100{\mathrm{ }}*{\mathrm{ }}[({A_{Cy3B,570}}/{\varepsilon_{Cy3B,570}})/\left( {{\rm concentration}\;{\mathrm{ }}{\rm of}\;{\mathrm{ }}{\rm product}} \right)]{\mathrm{ }}\% .
\end{eqnarray*}

\begin{eqnarray*}
{\rm Alexa647}\;{\mathrm{ }}{\rm labelling}\;{\mathrm{ }}{\rm efficiency}{\mathrm{ }} &=& {\mathrm{ }}100{\mathrm{ }}*{\mathrm{ }}\left[({A_{Alexa647,665}}/{\varepsilon_{Alexa647,665}})/\right.\\ &&\left.\left( {{\rm concentration}\;{\mathrm{ }}{\rm of}\;{\mathrm{ }}{\rm product}} \right)\right]{\mathrm{ }}\% ,
\end{eqnarray*}

where A_280_ is the measured absorbance at 280 nm, A_Cy3B,570_ is the measured absorbance of Cy3B (570 nm), A_Alexa647,665_ is the measured absorbance of Alexa647 (665 nm), ϵ_P,280_ is the molar extinction coefficient of σ^70^ at 280 nm (39 760 M^-1^ cm^-1^), ϵ_Cy3B,280_ is the molar extinction coefficient of Cy3B (10 400 M^-1^ cm^-1^) at 280 nm, ϵ_Alexa647,280_ is the molar extinction coefficient of Alexa647 at 280 nm (7350 M^-1^ cm^-1^), ϵ_Cy3B,570_ is the extinction coefficient of Cy3B at 570 nm (130 000 M^-1^ cm^-1^), and ϵ_Alexa647,665_ is the extinction coefficient of Alexa647 at 665 nm (245 000 M^-1^ cm^-1^). Labelling efficiencies were ∼88% for Cy3B and ∼75% for Alexa647.

### RNAP core enzyme

Hexahistidine-tagged *E. coli* RNAP core enzyme was prepared using co-expressed genes encoding RNAP β', β, α, and ω subunits to afford an RNAP core enzyme as follows: Single colonies of *E. coli* strain BL21(DE3) (Millipore) co-transformed with plasmid pEcABC-His_6_ [29] and plasmid pCDFω [30] were used to inoculate 20 ml LB broth containing 100 μg/ml ampicillin and 50 μg/ml kanamycin. Cultures were incubated for 16 h at 37°C with shaking. Culture aliquots (2 × 10 ml) were used to inoculate LB broth (2 × 1 L) containing 100 μg/ml ampicillin and 50 μg/ml kanamycin; cultures were incubated at 37°C with shaking until OD_600_ = 0.6; IPTG was added to 1 mM; and cultures were further incubated for 16 h at 16°C with shaking. Cells were harvested by centrifugation (4000 x *g*; 20 min at 4°C), resuspended in 20 ml buffer C (10 mM Tris–HCl, pH 7.9, 200 mM NaCl, and 5% glycerol), and lysed using an EmulsiFlex-C5 cell disrupter (Avestin). The lysate was cleared by centrifugation (20 000 x *g*; 30 min at 4°C), precipitated with polyethyleneimine (Sigma–Aldrich) as in [31], and precipitated with ammonium sulphate as in [31]. The precipitate was dissolved in 30 ml buffer C and loaded onto a 5 ml column of Ni-NTA-agarose (Qiagen) pre-equilibrated in buffer C, and the column was washed with 50 ml buffer C containing 10 mM imidazole and eluted with 25 ml buffer C containing 200 mM imidazole. The sample was further purified by anion-exchange chromatography on Mono Q 10/100 GL (GE Healthcare; 160 ml linear gradient of 300–500 mM NaCl in 10 mM Tris–HCl, pH 7.9, 0.1 mM EDTA, and 5% glycerol; flow rate = 2 ml/min). Fractions containing hexahistidine-tagged *E. coli* RNAP core enzyme were pooled, concentrated to ∼1 mg/ml using 30 kDa MWCO Amicon Ultra-15 centrifugal ultrafilters (EMD Millipore), and stored in aliquots at −80°C.

### DL RNAP-σ^70^

DL RNAP-σ^70^ was formed by incubating hexahistidine-tagged RNAP core enzyme with doubly labelled σ^70^ derivative in the ratio 1:2 in KG7 buffer (40 mM HEPES-NaOH, pH 7.0, 100 mM potassium glutamate, 10 mM MgCl_2_, 1 mM dithiothreitol, and 5% glycerol) at 30°C for 30 min and then on ice for 12 h.

### Nucleic acids

Lyophilized oligodeoxyribonucleotides (BiomersGmBH and IDT) were dissolved in nuclease-free water (Ambion) to a final concentration of 100 mM and stored at −20°C. For experiments involving DL RNAP-σ^70^, non-template oligodeoxyribonucleotides contained a 5′-biotinylated end. For experiments involving fluorescent-labelled oligodeoxyribonucleotides, non-template oligodeoxyribonucleotides were labelled with Cy3B and template oligodeoxyribonucleotides with Alexa647 in the positions stated (Supplementary Table S1).

Double-stranded DNA (dsDNA) was prepared as follows: Non-template oligodeoxyribonucleotides (1 μM) and template oligodeoxyribonucleotides (1.1 μM for annealing with biotinylated oligodeoxyribonucleotides and 1 μM for annealing with non-biotinylated oligodeoxyribonucleotides) in 50 μl 10 mM Tris–HCl, pH 7.9, and 0.2 M NaCl were heated for 5 min at 95°C and then cooled to 25°C in 2°C steps with 1 min per step in a thermal cycler (Applied Biosystems). The resulting dsDNA (∼1 μM) was stored at −20°C.

### Small molecules

NTPs (New England Biolabs) and dinucleotides (Trilink, Biolog LSI GmbH) were diluted in nuclease-free water (Ambion) to a final concentration of 100 mM or 10 mM and stored at −20°C.

### RP_o_

For experiments involving DL RNAP-σ^70^, RP_o_ was formed as follows: 15 nM DL RNAP-σ^70^ was incubated with 10 nM biotinylated double-stranded promoter DNA in 10 μl KG7 (40 mM HEPES-NaOH, pH 7.0, 100 mM potassium glutamate, 10 mM MgCl_2_, 1 mM dithiothreitol, and 5% glycerol) at 37°C for 20 min. For experiments involving double fluorescent-labelled dsDNA (ds), RP_o_ concentrations of 10 nM hexahistidine-tagged RNAP holoenzyme and 30 nM double-labelled dsDNA were used.

### *In vitro* transcription assays

*In vitro* transcription assays contained 15 ng/μL linear DNA template, an NTP mix (200 μM ATP/GTP/CTP, 10 μM UTP, and 2 μCi [α-32P] UTP), and 1.5 μM bovine serum albumin in transcription buffer (40 mM Tris–acetate, pH 7.9, 100 mM KCl, and 10 mM MgCl_2_). Reactions were initiated by the addition of wild-type RNAP-σ^70^ or DL RNAP-σ^70^, with a final concentration of 0.05 μM. Reactions were run for 10 min at 37°C before an equal volume of STOP solution (95% v/v deionized formamide, 7M urea, 20 mM EDTA, 0.05% bromophenol blue, and 0.05% xylene cyanol) was added. Reactions were resolved on an 8% sequencing gel before being exposed to a Bio-Rad phosphoscreen for 48 h. Images were visualized using the Amersham Typhoon biomolecular imager, and analysis was conducted using Image Lab (version 6.1.0).

### smFRET using TIRF-ALEX: sample preparation

Observation chambers with biotin-PEG-passivated glass floors were functionalized with neutravidin (Sigma–Aldrich). For experiments involving double-labelled dsDNA, the glass was additionally treated with biotinylated anti-hexahistidine monoclonal antibody (Penta-His Biotin Conjugate; Qiagen) as in [23, 32].

**Figure 2.**
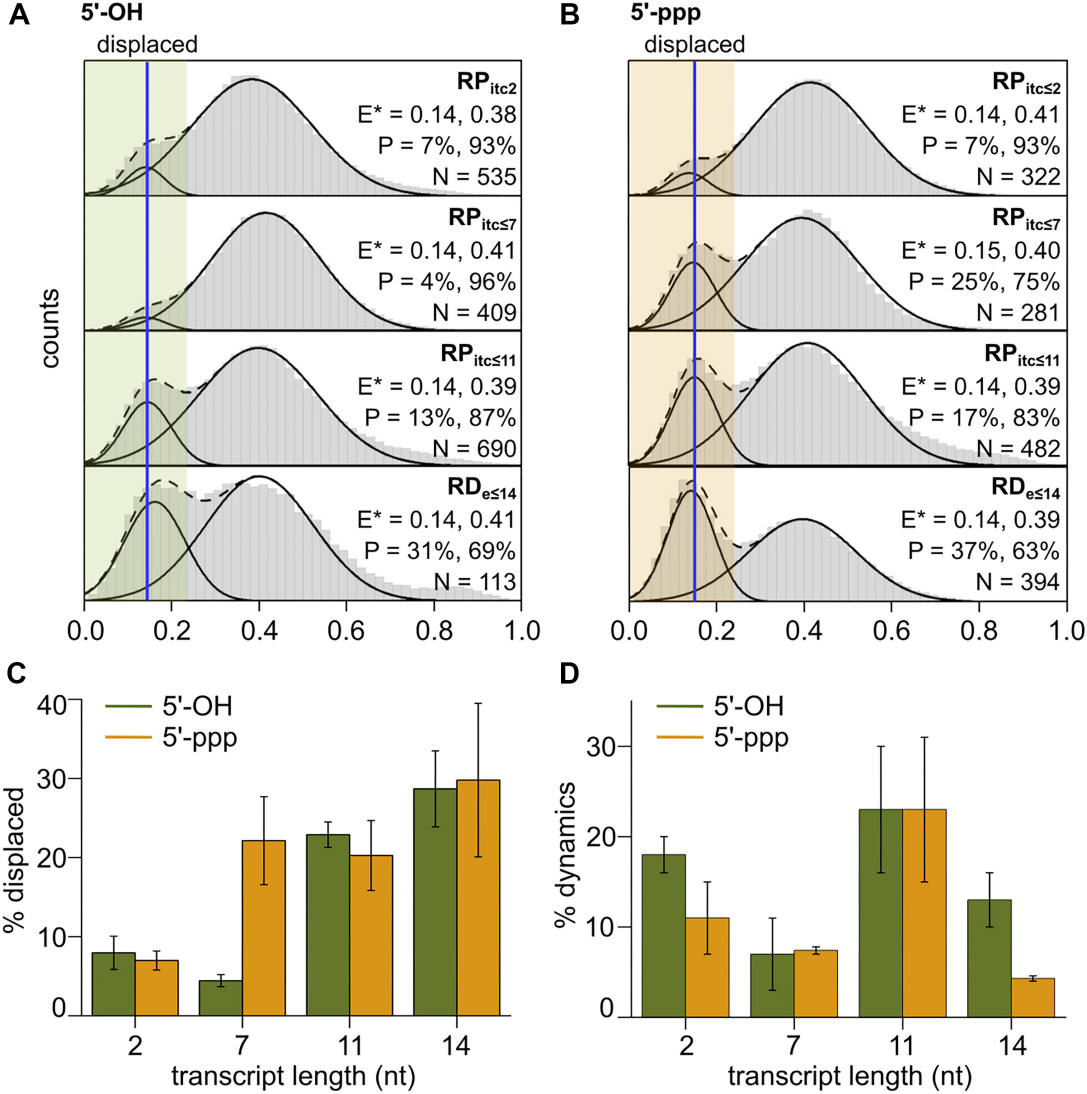
σ-finger displacement is affected by the 5′-end of RNA. (**A**) E* histograms of the σ-finger at stages of transcription involving the lacCONS promoter and RNA with a 5′-hydroxyl end (2, 7, 11, and 14 nucleotides in length). Estimations of mean E* values and occupation probabilities are shown for the displaced (blue line, green highlighting) and pre-displaced populations. (**B**) E* histograms of the σ-finger at stages of transcription involving the lacCONS promoter and RNA with a 5′-triphosphate end (2, 7, 11, and 14 nucleotides in length). Estimates of mean E* values and occupation probabilities are shown for the displaced (blue line, orange highlighting) and pre-displaced populations. (**C**) Occupation probabilities of the displaced σ-finger conformation with transcript length for RNA with a 5′-hydroxyl (green line) and a 5′-triphosphate (orange line) end. Error bars are calculated from the standard deviation in the mean between different subsets and repeats of the experiment. (**D**) Percentage of dynamic molecules with transcript length for RNA with a 5′-hydroxyl (green line) and a 5′-triphosphate (orange line) end. Error bars are calculated from the standard deviation in the mean between different subsets and repeats of the datasets.

**Figure 3.**
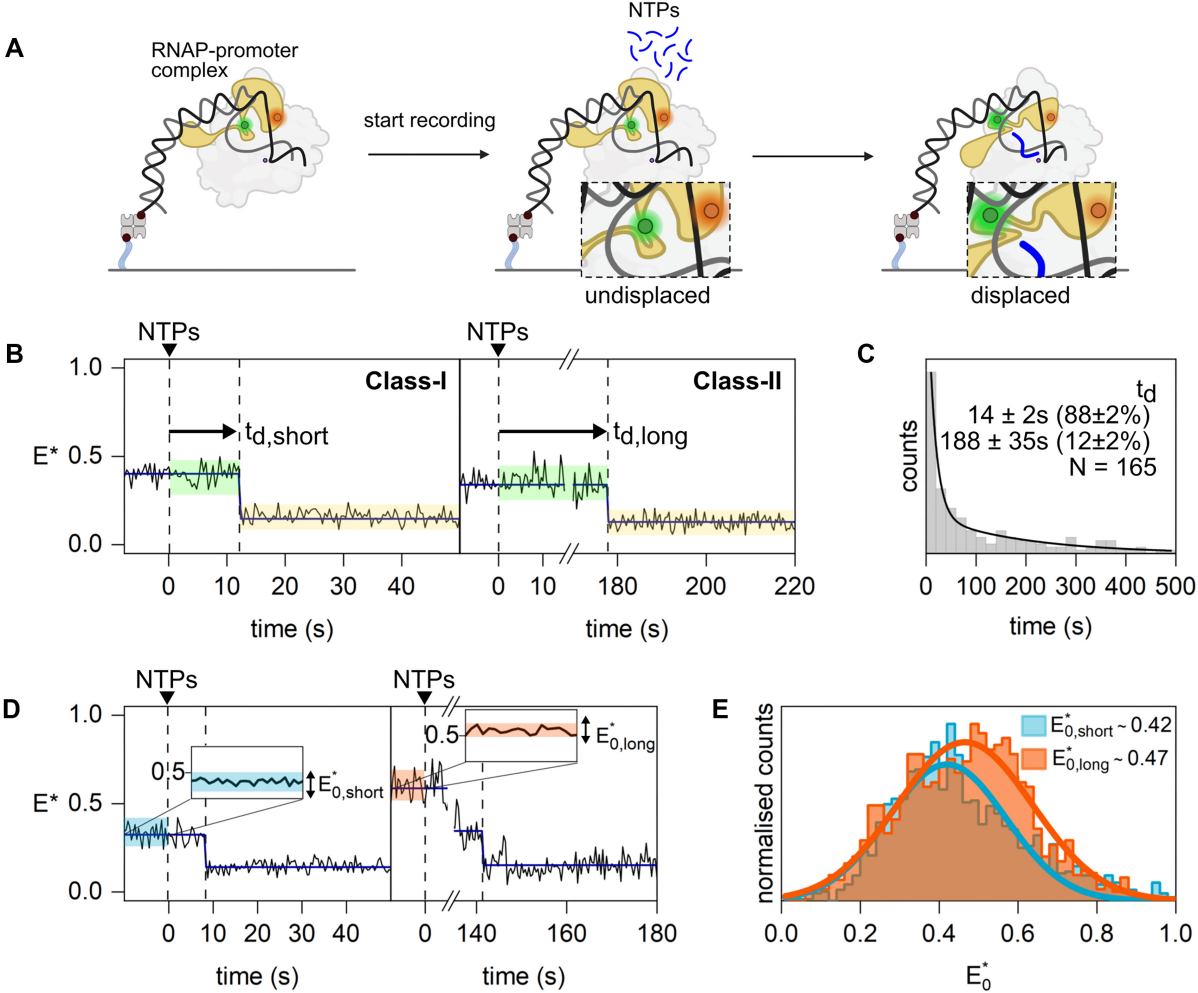
Real-time experiments monitoring the σ-finger conformation in initial transcription complexes containing the lacCONS promoter and RNA with a 5′-OH end. (**A**) Schematic of the real-time assay used to track σ-finger movements. (**B**) Example E*-time trajectories of Class-I and Class-II molecules showing σ-finger displacement. The conformation between NTP addition and displacement is highlighted in green, and the conformation after displacement is highlighted in yellow. (**C**) σ-finger displacement time histogram fitted with a double-exponential decay (black line) showing that displacement occurs on heterogeneous timescales. (**D**) Example E*-time trajectories of Class-I and Class-II molecules. The time before NTP addition is highlighted blue and red for Class-I and Class-II molecules, respectively. (**E**) Histograms showing the σ-finger conformation before NTP addition of Class-I (E*_0, short_; blue) and Class-II (E*_0, long_; red) molecules. All panels were created in BioRender. Mazumder, A. (2025) https://BioRender.com/22tuag8.

**Figure 4.**
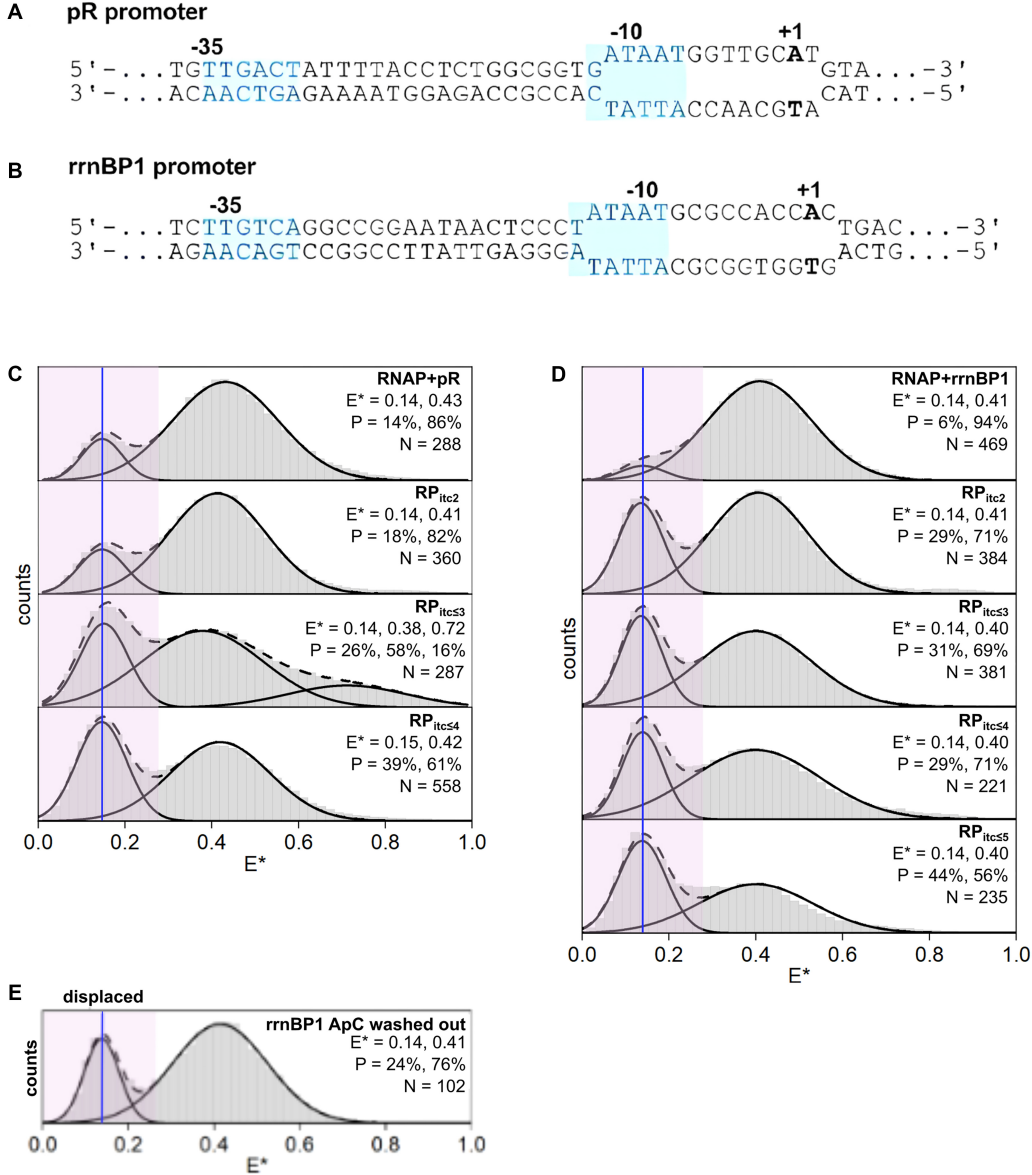
σ-finger displacement depends strongly on the promoter sequence. (**A**) pR DNA fragment used for the assay to track σ-finger movements. (**B**) rrnBP1 DNA fragment used for the assay to track σ-finger movements. (**C**) E* histograms of the σ-finger at stages of transcription involving the pR promoter and RNA with a 5′-hydroxyl end (0, 2, 3, 4, and 5 nt in length). Estimates of mean E* values and occupation probabilities are shown for the displaced (blue line, purple highlighting) and pre-displaced populations. (**D**) E* histograms of the σ-finger at stages of transcription involving the rrnBP1 promoter and RNA with a 5′-hydroxyl end (0, 2, 3, and 4 nt in length). Estimations of mean E* values and occupation probabilities are shown for the displaced (blue line, purple highlighting) and pre-displaced populations. (**E**) E* histogram of the σ-finger conformation involving the rrnBP1 promoter after incubation and removal of ApC.

**Figure 5.**
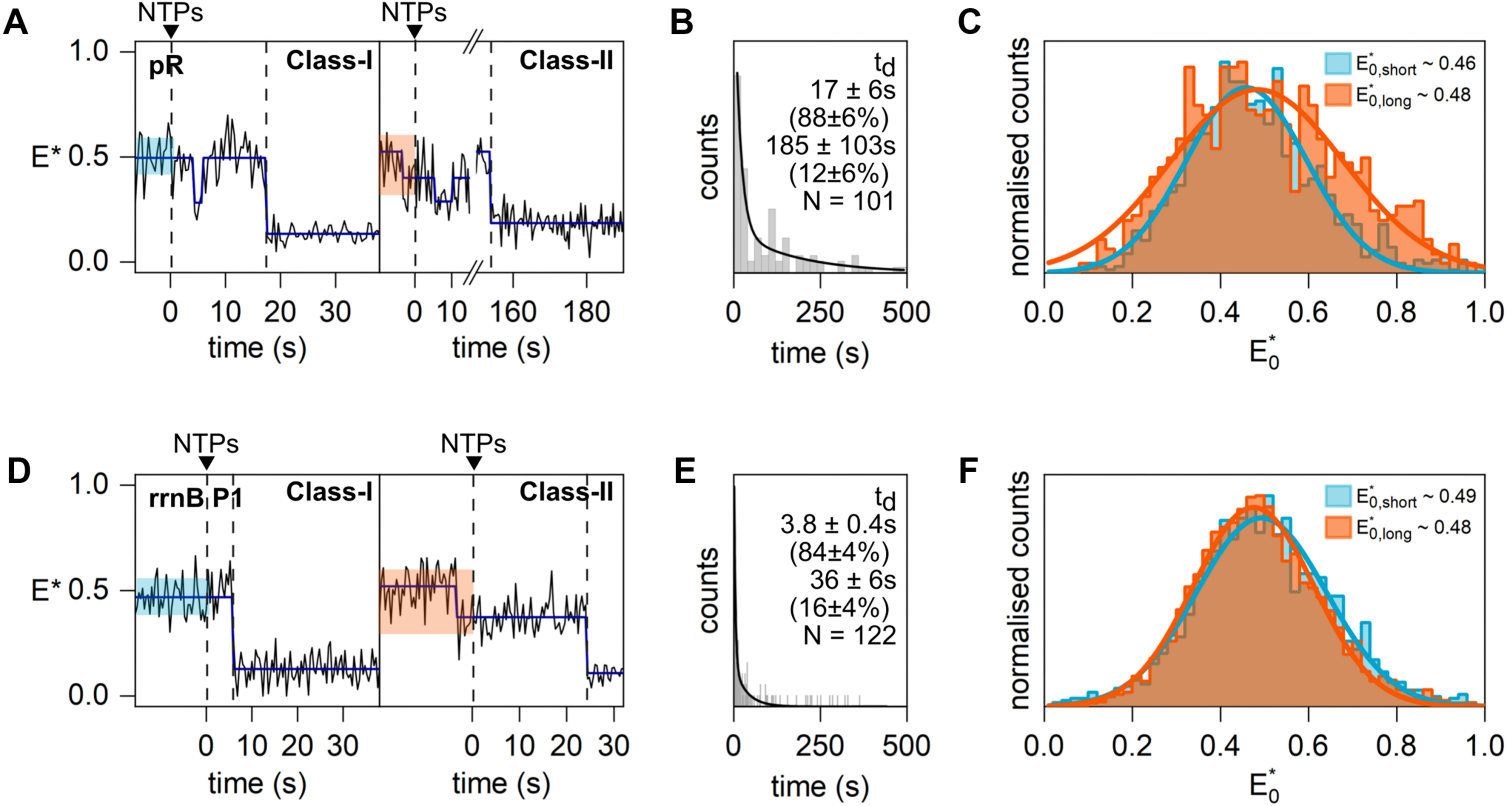
Real-time experiments monitoring the σ-finger conformation in initial transcription complexes containing the pR (**A**–**C**) and rrnBP1 (**D**–**F**) promoter and RNA with a 5′-OH end. (A, D) Example E*-time trajectories of Class-I and Class-II molecules showing σ-finger displacement. The conformation before NTP addition and displacement is highlighted blue and red for Class-I and Class-II molecules, respectively. (B, E) σ-finger displacement time histogram fitted with a double-exponential decay (black line) showing that displacement occurs on heterogeneous timescales. (C, F) Histograms showing the σ-finger conformation before NTP addition of Class-I (E*_0, short_; blue) and Class-II (E*_0, long_; red) molecules. All panels were created in BioRender. Mazumder, A. (2025) https://BioRender.com/22tuag8.

For experiments involving DL RNAP-σ^70^ in Figs 1–5, Supplementary Figs S5, S6, S8–S10, and S12, RP_o_ complexes containing biotinylated non-template strands were immobilized as follows: 50 μl of 0.1 nM double fluorescent-labelled RP_o_ in KG7 were added to the observation chamber and incubated for 10–30 s at 22°C. Wells were then washed with 3 × 50 μl KG7. For experiments involving double-labelled dsDNA and the DL RNAP-σ^70^ holoenzyme (Supplementary Figs S4, S7, and S13), complexes containing hexahistidine tags were immobilized in a similar fashion but incubated for 2–4 min in observation chambers that had been additionally treated with biotinylated anti-hexahistidine monoclonal antibody.

For experiments in Figs 2 (except RP_itc2_), 4 (except RP_o_ and RP_itc2_), Supplementary Figs S5, S7, and S13, and preformed RP_o_, they were immobilized as described previously, and a 50 μl solution of the specified combination of NTPs (100 μM) and dinucleotides (500 μM) in KG7 was added and incubated in the observation well for 5 min at 22°C. Observation wells were then washed with 5 × 50 μl KG7 and 50 μl imaging buffer [KG7 containing 2 mM Trolox (Sigma–Aldrich), 1 mg/ml glucose oxidase (Sigma–Aldrich), 40 μg/ml catalase (from bovine liver C30; Sigma–Aldrich), and 8 mM D-glucose] added. Data were then acquired. For RP_o_ experiments in Fig. 4 and Supplementary Fig. S12, preformed RP_o_ were immobilized; subsequently, 50 μl of imaging buffer was added to the well, and the well was imaged. For RP_itc2_ experiments in Figs 1D, 2 (RP_itc2_), 4 (RP_itc2_), Supplementary Figs S6 and S13, preformed RP_o_ were immobilized and 50 μl of imaging buffer supplemented with 500 μM of the relevant dinucleotide and imaged. For the experiment in Fig. 4E, preformed RP_o_ was immobilized and 500 μM ApC was added to the well and incubated for 5 min. The well was then washed with 5 × 50 μl KG7, 50 μl imaging buffer was added, and the well was imaged.

For real-time experiments with the lacCONS promoter DNA in Fig. 3 and Supplementary Figs S8–S10, RP_o_ was prepared as before, and 40 μl of imaging buffer supplemented with 625 μM dinucleotide (ApA or pppApA) was added to the well and incubated for 2 min at 22°C. Data acquisition was started and a 10 μl aliquot of 5 mM GTP/UTP/ATP in imaging buffer was added into the well and mixed. Final concentrations were 500 μM for the dinucleotide and 1 mM for the NTPs.

For real-time experiments involving the pR promoter in Fig. 5A–C, experiments were performed in a similar fashion with RP_o_ complexes prepared as earlier. An aliquot of 37.5 μl imaging buffer was added to the well. Data acquisition was then started and a 12.5 μl aliquot of 2 mM ApU, and 4 mM GTP/UTP/ATP was added into the well and mixed. Final concentrations were 500 μM of ApU and 1 mM NTPs. The same method was used for real-time experiments involving the rrnBP1 promoter in Fig. 5D–F but with final concentration of 500 μM ApC and 1 mM GTP/UTP/ATP/CTP instead.

For experiments in Supplementary Fig. S7, RP_o_ complexes were prepared as above, and a 50 μl solution of 500 μM ApA and 100 μM UTP/GTP/ATP in KG7 was incubated in the well for 5 min at 22°C to form RP_itc≤11_. Wells were washed with 5 × 50 μl KG7 and 50 μl imaging buffer was added. Data were then acquired for RP_itc≤11_. Immediately after the imaging, wells were washed with 5 × 50 μl KG7, and a 50 μl aliquot of 1 mM GTP/CTP was incubated into the well for 5 min at 22°C to form RD_e≤14_. Data were then acquired for RD_e≤14_.

### smFRET using TIRF-ALEX: data collection

smFRET experimental data were collected using a custom-built objective-type total internal reflection fluorescence (TIRF) microscope. Light from green (532 nm, Cobolt) and red (635 nm, Coherent) lasers was combined using a dichroic mirror and coupled into single-mode fibre. The output was focused onto the back focal plane of a 100× oil-immersion objective (NA 1.4, Olympus) and laterally displaced from the optical axis such that the incident angle at the oil-glass interface of the observation chamber exceeded the critical angle and created an evanescent illumination wave. Alternating-laser excitation (ALEX) was achieved through an acousto-optical modulator. A dichroic mirror (545 nm/650 nm, Semrock) and emission filters (545 nm LP Chroma and 633/25 nm notch, Semrock) separated the fluorescence emission from the excitation. The fluorescence emission was then focused on a slit and spectrally separated using a dichroic mirror (630 nm DLRP, Omega) into two channels focused onto an EMCCD (iXon 897, Andor). A motorized x/y-scanning stage (MS-2000, ASI) was used to mount the observation chambers.

All data acquisition was carried out at 22°C using ALEX-TIRF. For experiments in Figs 1C, 2A and B, and 4, data were collected for 50 s at a frame rate of 100 ms/frame (50 ms ALEX) using laser powers of 0.7 mW (532 nm laser) and 0.3 mW (635 nm laser). For experiments in Supplementary Figs S7 and S13, data were collected for 50 s at a frame rate of 100 ms/frame (50 ms ALEX) using laser power of 0.6 mW (532 nm laser) and 0.2 mW (635 nm laser). For experiments in Figs 3 and 5, data were collected for 500 s at a frame rate of 400 ms/frame.

### smFRET using TIRF-ALEX: data collection

Background-corrected fluorescence-emission intensity versus time trajectories for donor emission upon donor excitation (I_DD_), acceptor emission upon donor excitation (I_DA_), and acceptor emission upon acceptor excitation (I_AA_) were extracted using software package Twotone-ALEX [33]. Intensity versus time trajectories were manually inspected to exclude trajectories exhibiting I_DD_< 100 or > 1500 counts, I_AA_< 100 or > 1000 counts, multi-step donor or acceptor photobleaching, and donor or acceptor photoblinking. Selected trajectories were then used to calculate trajectories of apparent FRET efficiencies (E*) and donor-acceptor stoichiometry (*S*) using equations:


\begin{eqnarray*}
E*{\mathrm{ }} = {\mathrm{ }}{I_{{\rm DA}}}/\left( {{I_{{\rm DD}}} + {I_{{\rm DA}}}} \right),
\end{eqnarray*}

\begin{eqnarray*}
S{\mathrm{ }} = {\mathrm{ }}\left( {{I_{{\rm DD}}} + {I_{{\rm DA}}}} \right)/\left( {{I_{{\rm DD}}} + {I_{{\rm DA}}} + {I_{{\rm AA}}}} \right).
\end{eqnarray*}

*S* values were used to distinguish between species containing both donor and acceptor fluorophores and species containing only one of either donor or acceptor fluorophores. E* histograms for species containing both donor and acceptor fluorophores were built and fitted to Gaussian distributions using Origin (Origin Lab) to provide subpopulation percentages and mean E* values as in Figs 1D, 2, 3E, 4C–E, 5C, and 5F, and Supplementary Figs S4 and S12. In Fig. 2C, the error in the low E* ‘displaced’ subpopulation percentage was calculated as follows: Datasets from experiments in Fig. 2A and B were split into their different individual experimental datasets, where it had been performed more than once (or into three subsets, where the experiment was only performed once). These data subsets were then individually plotted and fitted to Gaussian distributions to find a mean and standard deviation in the mean of the low-E* ‘displaced’ subpopulation percentage as plotted in Fig. 2C.

To identify any dynamics and their time scales, E*-time trajectories were analysed using Hidden Markov Modelling (HMM) as implemented in the software package ebFRET [33]. By maximizing the lower bound (a scoring parameter) [34, 35], a three-state model was chosen to fit any dynamic E*-time trajectories in experiments involving DL RNAP-σ^70^. For experiments in Figs 1E, 2, and Supplementary Figs S4–S6, dynamic molecules were defined as those with three or more transitions between different states. Fitted E* values from the trajectories classified into the three-state model were then plotted and fitted to Gaussian distributions in Origin to extract population percentages and a mean E* value for each state. Dwell times were extracted and plotted as histograms in Supplementary Figs S4–S6, which were then fit to single exponential decays in all cases, apart from the histogram in S6, where a peaked exponential decay was used for the low-FRET state. To estimate the error in the percentage of dynamic molecules shown in Fig. 2D, datasets from experiments in Fig. 2A and B were split into their different individual experimental datasets, where they had been performed more than once (or into three subsets, where the experiment was only performed once). The percentage of dynamic molecules for each data subset was then individually calculated to find a mean and standard deviation in the mean of the % of dynamic molecules as plotted in Fig. 2D.

For real-time experiments in Figs 3, 5, and Supplementary Figs S8–S10, all E*-time trajectories were analysed initially using HMM [34, 35] and then categorized into four groups as described in the main text. After categorization, HMM analysis was re-run on groups exhibiting displacement (4-state model to incorporate the displaced state) or dynamics (3-state model) separately. Displacement time histograms for σ-finger displacement (Figs 3C, 5B and 5E, and Supplementary Fig. S10A) were calculated by subtracting the time of nucleotide addition from the time of σ-finger displacement, taken from HMM analysis. The time for σ-finger displacement was then plotted as histograms and fit to double exponential decays in Origin, and the two time constants were extracted. For analysis in Figs 3E, 5C, and 5F and Supplementary Fig. S10B, E* values before nucleotide addition were extracted from traces exhibiting σ-finger displacement (Class-I and Class-II). These traces were classified into either exhibiting ‘short’ or ‘long’ finger displacement using a boundary calculated from the time at which the two components of the double exponential decay intersect. The E*-values for these two groups were then extracted, and a histogram was produced from binned values. These were then fit to single Gaussian distributions in Origin.

### Accurate FRET calculations

E* values were corrected for leakage of donor emission into the acceptor emission channel, direct excitation of the acceptor by the 532 nm laser, and different detection efficiencies of the donor and acceptor as described in [35]. The leakage factor (Lk) was calculated using E* values of donor-only molecules (E*_D-only_),


\begin{eqnarray*}
Lk = {\mathrm{\;}}\frac{{E_{{\rm D -} {\rm only}}^{\mathrm{*}}}}{{1 - E_{{\rm D -} {\rm only}}^{\mathrm{*}}}}.
\end{eqnarray*}

The direction excitation factor (*Dir*) was calculated using *S* values of acceptor-only molecules (*S*_A-only_):


\begin{eqnarray*}
Dir = {\mathrm{\;}}\frac{{{S_{{\rm A - only}}}}}{{1 - {S_{{\rm A - only}}}}}.
\end{eqnarray*}

The FRET proximity ratio (*E*_PR_) was calculated as:


\begin{eqnarray*}
{E_{{\rm PR}}} = {\mathrm{\;}}\frac{{1 - Dir{\mathrm{*}}\left( {\frac{{1 - S}}{S}} \right) - Lk{\mathrm{*}}\left( {\frac{{1 - {E^{\mathrm{*}}}}}{{{E^{\mathrm{*}}}}}} \right)}}{{\frac{{1 - {E^{\mathrm{*}}}}}{{{E^{\mathrm{*}}}}} + 1 - Dir{\mathrm{*}}\frac{{1 - S}}{S}}}.
\end{eqnarray*}

The detection factor, γ, was calculated by $\gamma = \;\frac{{\Delta {I_{DA}}}}{{\Delta {I_{DD}}}}$, where $\Delta {I_{DA}}$ and $\Delta {I_{DD}}$ are the changes in ${I_{DA}}$ and $\Delta {I_{DD}}$ upon acceptor photobleaching. The accurate FRET (*E*_a_) was then calculated by:


\begin{eqnarray*}
{E_a} = {\mathrm{\;}}\frac{{{E_{{\rm PR}}}}}{{\gamma - \left( {\gamma - 1} \right){\mathrm{*}}{E_{{\rm PR}}}}}.
\end{eqnarray*}

## Results

### Strategy for detecting σ-finger motions at the single-molecule level

For the detection of σ-finger motions by smFRET during initial transcription, we designed a doubly labelled hexahistidine-tagged RNAP-σ^70^ holoenzyme (DL RNAP-σ^70^), with one fluorescent probe attached to the σ-finger and another fluorescent probe attached to σR2 (σ^70^ residue 366)—a position that has been used to attach fluorescent probes without compromising RNAP function for ensemble fluorescence and smFRET studies on transcription initiation and promoter escape [25, 28, 36, 37]. Since the σ-finger is a small structural module that interacts with the growing RNA during initial transcription, the position of attachment of the fluorescent probe was carefully chosen to minimize any interference with RNAP function. Accessible volume (AV) modelling of fluorescent probes [27] attached to positions 511 to 521 of σ^70^ on high-resolution structures of initial transcribing complexes having a 3-, 4-, or 5-nt RNA revealed that a fluorescent probe placed at position 511, located at the base of the finger, should not interfere with σ-finger-RNA interactions (Supplementary Fig. S1B), since the AV cloud (denoting all possible orientations the attached probe can occupy) lies away from the tip of the finger and away from the path of the extending RNA. The distance between fluorescent probes Cy3B and Alexa647 placed, respectively, at positions 511 and 366 of σ^70^ was estimated to be in the 58–66 Å range (using PDB structures 4YLN, 7KHC, 7KHB, 7MKD, and 7MKE), with corresponding estimated FRET efficiencies (E) in the 0.30–0.55 range (Fig. 1B)—a range well suited for monitoring distance changes of ∼5–20 Å. We thus selected these two positions to generate a DL RNAP-σ^70^ construct (see the ‘Materials and methods’ section; Supplementary Fig. S2A). The labelling efficiencies of DL RNAP-σ^70^ were ∼88% and ∼75% for Alexa647 and Cy3B, respectively, while the transcriptional activity was ∼80% compared to the activity of unmodified RNAP-σ^70^ (Supplementary Fig. S2B and C), indicating that the attached fluorescent probes did not significantly affect RNAP assembly and RNAP function. Further, to ensure the incorporated dyes can rotate freely after attachment to the protein, we measured steady-state anisotropy values, which showed that both donor and acceptor probes attached to the doubly labelled RNAP reorient on the timescale of donor excited-state lifetime (Supplementary Fig. S2D) [38]. Importantly, this labelling scheme generates three types of doubly labelled RNAP: a donor–donor DL RNAP-σ^70^, an acceptor-acceptor DL RNAP-σ^70^, and a donor-acceptor DL RNAP-σ^70^. To separate the donor-acceptor DL RNAP-σ^70^ from the other species, we use an ALEX [39], which reports on the donor-acceptor stoichiometry of single molecules in addition to the FRET efficiency (see the ‘Materials and methods’ section).

To assess whether the attachment of a fluorescent probe at the base of the σ-finger or residue 366 affects RNAP function in any way, we performed bulk *in vitro* transcription reactions on the three promoters used in our study (lacCONS, pR, and rrnBP1). The results of these experiments showed that, in all three cases, similar levels of run-off transcripts were produced at a similar rate compared to the wild-type RNAP holoenzyme (Supplementary Fig. S3A). Further, to evaluate whether the presence of fluorescent probes at the base of the σ-finger alters the profile of the initiation pause observed for lacCONS (a pause observed after formation of a 6-mer RNA for lacCONS [23]), we performed *in vitro* transcription assays directing formation of a 7-mer RNA. Results revealed that the rate of accumulation of the 7-mer RNA using the DL RNAP-σ^70^ and a wild-type RNAP-σ^70^ were similar (Supplementary Fig. S3C). Taken together, these results establish that the introduction of a fluorescent probe at the base of the finger does not alter RNAP function during initial transcription and promoter escape.

### The σ-finger is mobile in the RNAP holoenzyme and in an RNAP-promoter complex

To assess σ-finger conformations in transcription initiation, we incubated DL RNAP-σ^70^ with a biotinylated consensus bacterial core promoter fragment (biotin-lacCONS; Supplementary Table S1 and Fig. 1D) to form heparin-resistant RNAP-promoter open complexes (RP_o_) using established protocols (see the ‘Materials and methods’ section), followed by immobilization of individual RP_o_ molecules to glass slides functionalized with neutravidin (Fig. 1C). Next, we added the initiating dinucleotide adenosine-5′-phosphoadenosine dinucleotide (ApA) to the immobilized RP_o_ molecules to form an initial transcribing complex (RP_itc2_) and carried out TIRF microscopy with ALEX experiments as described previously [35, 39]. The data were analysed to obtain apparent smFRET efficiencies (E*) from E*-versus-time trajectories of single RP_itc2_ complexes and to generate an E* histogram that reports on the distribution of σ-finger conformational states.

The E* histogram for RP_itc2_ was multimodal with two subpopulations (Fig. 1E, top right): a major subpopulation with mean E*∼0.38 (93%) and a minor subpopulation with mean E*∼0.14 (7%). Inspection of E*-versus-time trajectories (Fig. 1E, left) revealed a subpopulation of molecules (18%) that showed transitions between different E* values; analysis of E*-versus-time trajectories from these dynamic molecules using HMM [34, 35] identified three subpopulations with mean E* of ∼0.22 (17%), ∼0.37 (54%), and ∼0.59 (29%) and dwell times in the range of ∼0.6–1.0 s (Fig. 1E, bottom right).

Distance estimates corresponding to subpopulations with mean E* values of ∼0.37 and ∼0.59 (corresponding to distances of ∼58–66 Å) were in excellent agreement with those predicted from AV calculations on high-resolution structures of *E. coli* RNAP and RNAP-promoter complexes (65.9 ± 9.1 Å, 4YLN; 61.4 ± 8.2 Å, 7KHB; 64.7 ± 8.9 Å, 7MKD; and 62.7 ± 6.1 Å, 7MKE; 57.7 ± 4.3 Å, 7KHC: Supplementary Fig. S1A). In all these structures, the σ-finger occupies an in-cleft position capable of interacting with the template DNA strand loaded in the RNAP main channel. We thus assigned subpopulations with mean E* values of ∼0.37 and ∼0.59 to ‘in-cleft’ σ-finger conformations as visualized in the high-resolution structures. We note that the large variation in distances (and correspondingly in E* values) for the same σ-finger conformation originates in part from different positions of the σ^70^ residue 366 in the structures. On the other hand, we define subpopulations with mean E* values of ∼0.22 (distance ∼81 Å) to represent structural states where the σ-finger is positioned away from σR2 and suggest that these represent conformations where the σ-finger is away from the in-cleft position observed in the structures. A transition density plot generated from the HMM analysis revealed majority of the transitions took place between E* values representing the ‘in-cleft’ σ-finger subpopulation, with only rare transitions to the low E* subpopulation (Supplementary Fig. S6). We suggest that this structural state has not yet been captured in structural studies, likely due to high mobility of the σ-finger in this conformation.

In the next set of experiments, we measured apparent smFRET efficiencies (E*) of DL RNAP-σ^70^ molecules immobilized via a hexahistidine tag on a glass slide functionalized with anti-hexahistidine tag antibody (Supplementary Fig. S4A). As for RP_itc2_, the overall E* histogram for DL RNAP-σ^70^ revealed a multimodal distribution, with populations centred around mean E* values of ∼0.15 (10%), ∼0.37 (72%), and ∼0.69 (18%) (Supplementary Fig. S4B). A visual inspection of the E*-versus-time trajectories revealed a class of molecules (28%) that exhibited transitions between different FRET states and were classified as dynamic molecules, within which we identified three subpopulations having mean E* values of ∼0.22 (28%), ∼0.45 (54%), and ∼0.70 (18%), all with dwell times in these conformational states on time scales of ∼0.5–0.8 s (Supplementary Fig. S4C). Further, a transition density plot generated from the HMM analysis revealed transitions between all conformational subpopulations, implying significant σ-finger conformational dynamics in these complexes (Supplementary Fig. S4).

### σ-finger displacement during initial transcription: consensus bacterial promoter (lacCONS)

We then assessed σ-finger conformations in primer-dependent initial transcription for synthesis of RNAs up to 11 nt in length. Initial transcribing complexes (RP_itc_) were formed by adding subsets of nucleotides to RP_itc2_ complexes prepared using the lacCONS construct (Supplementary Table S1). Use of subsets directs transcription up to a desired maximum RNA length when subsets of NTPs are used—a practice that we had established in previous studies using bulk *in vitro* transcription assays [23]; further, use of ApA dinucleotide as a primer led to synthesis of RNAs with a 5′-OH group.

For complexes making RNAs of up to 7 nt in length prepared using a mixture of ApA, UTP, and GTP (RP_itc≤7_), the E* histogram showed almost exclusively the presence of the subpopulation corresponding to an ‘in-cleft’ σ-finger conformation (∼93%–100%; Fig. 2A). Significantly, the E* time trajectories showed few dynamics (<10% of dynamic molecules; Fig. 2D), indicating formation of a stabilized conformation for the σ-finger module during this stage of initial transcription. The lack of significant structural heterogeneity and dynamics for our construct (which probes the motions of the *base* of the σ-finger; Supplementary Fig. S1B) indicates that it forms a stable structural state for the base of the finger when <8-mer RNA is synthesized.

In contrast, further RNA extension to 11 nt led to increased abundance for low-FRET species [E*∼0.14 (18%) for RP_itc≤11_; Fig. 2A], indicating that, for a significant fraction of molecules, synthesis of an 11-mer RNA results in σ-finger displacement from an ‘in-cleft’ conformation to a conformation positioned away from σR2, which we assign to a ‘displaced’ σ-finger conformation. Notably, analysis of E* time trajectories for RP_itc≤11_ revealed a much higher fraction of dynamic molecules (21%, Fig. 2D), with transitions between three subpopulations centred around E*∼0.23, 0.42, and 0.64, occurring on timescales of ∼1.0–1.4 s (Supplementary Fig. S5). From the transition density plot generated following HMM analysis, we identified that transitions involving the E*∼0.23 subpopulation are rare, and most transitions occur between the other two conformational subpopulations (Supplementary Fig. S5D). The increased mobility of the σ-finger for a large fraction of molecules during late stages of initial transcription for lacCONS (<11-nt-long RNA) as compared to earlier stages (<7-nt RNA) may originate either from a loss of contact with the scrunched DNA–RNA hybrid resulting in motion of the s-finger between different in-cleft structural states or due to motions of the entire σ-finger bound to the DNA–RNA hybrid resulting from scrunching–unscrunching of the transcription bubble as observed in earlier single-molecule studies [32]. If the resulting dynamics were indeed similar to that observed in the early stages of initial transcription, then this result would suggest that, at the stage of finger displacement, the σ-finger breaks its contact with the DNA–RNA hybrid and becomes mobile, exploring several conformational states. However, a dwell-time analysis for subpopulations with a mean E*∼0.23 revealed a peaked distribution for dwell times corresponding to different conformational states populated in RP_itc≤11;_ this distribution shape is in sharp contrast to exponential distributions observed previously for RP_itc2_ (Supplementary Fig. S6). A peaked dwell-time distribution suggests a conformational change involving more than one step, indicating that the σ-finger motions in these late stages of initial transcription correlate with the previously observed dynamics of the transcription bubble [32]. Further extension to a 14-mer RNA (RD_e≤14_) resulted in an E* histogram with a subpopulation around ∼0.14 (31%) and a reduction in the fraction of dynamic trajectories (to 13%; Fig. 2D). This reduction likely suggests progression out of the late stages of initial transcription and promoter escape to form an early elongation complex that still retains σ^70^.

To determine the status of the transcription bubble for this promoter during initial transcription, we performed similar experiments using a previously reported lacCONS construct with Cy3 attached to +2 position (lacCONS-[+2-Cy3]) of the non-template strand. Previous studies have shown that RNAP promoter complexes prepared using this construct show a rapid decrease in Cy3 fluorescence intensity upon promoter escape [40, 41]. We performed experiments using subsets of NTPs to generate initial transcribing complexes with 2-mer RNA (added ApU), 7-mer RNA (added ApU, UTP, and GTP), and 11-mer RNA (added ApU, UTP, GTP, and ATP). None of these reactions exhibited a significant fluorescence decrease as is observed when all NTPs are added (Supplementary Fig. S7). We conclude, therefore, that for this promoter, the point of escape lies beyond synthesis of an 11-mer RNA, and that for the lacCONS promoter the base of the σ-finger is displaced during initial transcription and before promoter escape.

### Point of σ-finger displacement depends on the functional group at the 5′-end of RNA

We then assessed σ-finger conformations in primer-independent transcription, which results in synthesis of RNAs with a 5′-triphosphate (5′-ppp), a chemical moiety with greater steric bulk and additional negative charges compared to a 5′-OH, and thus with potential for different interactions with the negatively charged σ-finger. We performed experiments similar to those in the previous section but in the absence of a dinucleotide primer and measured σ-finger conformational dynamics for complexes capable of synthesizing RNAs from up to 2 nt in length (ppp-RP_itc≤2_), 7 nt in length (ppp-RP_itc≤7_), and 11 nt in length (ppp-RP_itc≤11_). Analysis of E* distributions for ppp-RP_itc≤2_ showed results similar to those obtained for prime-dependent transcription initiation (Fig. 2B). However, further extension to a 7-mer RNA resulted in an E* histogram with an increased fraction of the subpopulation with E*∼0.15 (25%; compared to 4% for a 7-mer with a 5′-OH end). Similar results were obtained for complexes capable of synthesizing up to an 11-mer RNA, with occupancies in the low FRET state around 17% (Fig. 2B and C, orange line); additionally, a significant fraction of E* time trajectories (∼23%) for these complexes show dynamic behaviour (Fig. 2D, orange line), analogous to results obtained for RP_itc≤11_ in the primer-dependent transcription initiation. Our results indicate that, in primer-independent initiation, the unfavourable interactions between the bulky, negatively charged triphosphate group and the σ-finger (which has a negative net charge) result in displacement at a much shorter RNA length compared to primer-dependent initiation.

### Kinetics of σ-finger displacement during initial transcription

To observe the kinetics of σ-finger displacement during initial transcription, we performed real-time RNA synthesis up to the point of promoter escape during the recording of our single-molecule movies. Specifically, we preformed RP_o_ with DL RNAP-σ^70^ and lacCONS promoter DNA fragments at 37°C, immobilized RP_o_ molecules (as in Fig. 1C), formed RP _itc2_ on glass, started recording of the movie, and added a subset of NTPs directing transcription up to the formation of a stable elongation complex (for lacCONS, this corresponds to synthesis of a 14-nt RNA; Fig. 3A). To compare the kinetics of σ-finger displacement during primer-dependent transcription initiation with those of primer-independent transcription initiation, RP_itc2_ was formed with either an ApA dinucleotide primer or a pppApA dinucleotide primer, respectively. Movies were analysed to extract E* time trajectories and were fit using HMM to extract the time between nucleotide addition and σ-finger displacement (see the ‘Materials and methods’ section), a time that we will refer to as displacement time t_d_.

A visual examination of the E* time trajectories (Supplementary Fig. S8) obtained in the presence of ApA revealed several classes of molecules. Class-I molecules showed a step decrease in E* to a stable state around E*∼0.15, which corresponds to the ‘displaced’ σ-finger; this transition occurred on a short timescale (Fig. 3B, left). In contrast, Class-II molecules transitioned to a stable ‘displaced’ σ-finger conformation on a long timescale (Fig. 3B, right). Class-I and Class-II molecules made up 25% of all molecules. Class-III molecules (44% of all molecules; Supplementary Fig. S8) showed no transition to an E*-state characteristic of the ‘displaced’ σ-finger but exhibited transitions between other E* states. An inspection of E*-versus-time trajectories for molecules in an experiment with preformed elongation complexes (RD_e≤14_) revealed fewer dynamic molecules (13%; Fig. 2D), indicating that, for some of the dynamic molecules observed in the real-time experiments, the σ-finger is displaced at later time points and is missed because of premature termination of the time-trajectory due to bleaching. Finally, Class-IV molecules (31% of all molecules, Supplementary Fig. S8C) showed no transitions and likely consist of either inactive complexes or complexes that remain at early stages of initial transcription.

To estimate the displacement time t_d_, we combined molecules from Class-I and Class-II, binned them into a dwell-time histogram, and fitted the distribution with a biexponential decay (Fig. 3C). Results showed two types of displacement events, a fast one, t_d,short_ ∼14 s (accounting for ∼90% of events), and a slow one, t_d,long_ ∼190 s (accounting for ∼10% of events).

Similar experiments with pppApA revealed E*-versus-time trajectories that contained all four classes of molecules seen in the absence of the 5′-triphosphate group on the RNA, with abundances of 41% for Class-I and Class-II, 27% for Class-III, and 32% for Class-IV (Supplementary Fig. S9). Analysis of displacement times from Class-I and Class-II also showed some heterogeneity in displacement times, with t_d,short_ ∼1.4 s (∼97% of the events) and t_d,long_ ∼70 s (∼3% of the events; Supplementary Fig. S10A). Strikingly, the displacement events for Classes I and II for transcription with an RNA having a 5′-triphosphate occurred at much faster timescales compared to transcription with an RNA having a 5′-OH. Together with the observation that the σ-finger is displaced at much shorter RNA lengths for primer-independent initiation (5-nt RNA) compared to primer-dependent initiation (10-nt RNA), this result points to a much lower energy barrier for σ-finger displacement when a triphosphate is present at the 5′-end of the growing RNA, likely resulting from unfavourable interactions between the largely negatively charged σ-finger module (due to the presence of a stretch of four acidic residues in the σ-finger: D513, D514, E515, and D516) and the bulky, negatively charged triphosphate group.

We then explored the intriguing observation of molecules with very different displacement kinetics. Since the real-time reaction starts with the addition of NTPs to immobilized RP _itc2_ complexes, we hypothesized that the different kinetics may be associated with conformationally different RP _itc2_ molecules and, especially, with different conformations of the σ-finger, which plays key roles in transcription initiation and promoter escape. To test this hypothesis, we analysed the σ-finger conformations before NTP addition for two types of molecules: those showing fast displacement kinetics (‘fast-displacers’, with t_d_< 31 s for primer-dependent initiation and t_d_< 4 s for primer-independent initiation) and those showing slow displacement kinetics (‘slow-displacers’, with t_d_> 31 s for primer-dependent initiation and t_d_> 4 s for primer-independent initiation). This analysis revealed distinctly different E* histograms for the two types of species on lacCONS: molecules with a wide E* distribution centred around ∼0.42 for fast displacers and molecules with a wide E* distribution centred around ∼0.47 for slow displacers (Fig. 3D and E). Similar results on lacCONS were obtained for both primer-dependent (5′-OH RNA) and primer-independent transcription initiation (5′-ppp RNA) (Fig. 3E and Supplementary Fig. S10B).

### σ-finger displacement during initial transcription: a phage λ promoter (pR)

We then examined the profile of σ-finger displacement in different promoters, since it is well known that initial transcription and promoter escape are heavily dependent on the promoter and initially transcribed sequences [42, 43]. We performed analogous experiments with two widely studied and naturally occurring bacterial promoters, the λ pR and the rrnBP1 (Fig. 4A and B, Supplementary Fig. S14), which exhibit significantly different behaviour during initial transcription. The pR promoter is characterized by formation of a very stable RP_o_^26^ (lifetime > 10^4^ s), a 13-bp unwound bubble, and a transcription start site seven bases downstream of the −10 element. The pR promoter directs accumulation of short RNA products (abortive initiation) during initial transcription and escape from the promoter at an RNA length of 11 nt [26, 44]. Structural studies of RP_o_ formed at the pR have shown that the σ-finger module protrudes into the active site cleft to contact the single-stranded template DNA and is positioned to clash with a growing RNA chain of 4–6 nt in length [19] (Supplementary Fig. S11).

Experiments performed on an RP_o_ formed on a pR promoter fragment showed a major subpopulation with mean E*∼0.43 (86%) representative of an in-cleft σ-finger and a minor conformation with mean E*∼0.14 (14%) representative of a displaced σ-finger (Fig. 4C); these results were similar to those obtained on an RP_o_ formed on the lacCONS promoter (Supplementary Fig. S12). Measurements performed with initial transcribing complexes containing varying lengths of RNA first revealed that the abundance of the subpopulation corresponding to the displaced σ-finger was similar for RP_itc≤2_ (18%). Surprisingly, however, the displaced σ-finger conformation significantly increased for RP_itc≤3_ (26%) and RP_itc≤4_ (39%) (Fig. 4C), indicating that displacement occurs after synthesis of a very short RNA (just a 3-mer) and well before the RNA reaches sufficient length to clash with the σ-finger for the pR promoter (∼5 nt). Notably, for RP_itc≤ 3_, an additional subpopulation appears at E* ∼0.72 (16%). These conformations may represent an in-cleft σ-finger conformation where the σ-finger contact with the template DNA strand is broken, causing the finger to adopt either an in-cleft conformation closer to σR2 or a conformation associated with the σ-finger bound to an RNA–DNA hybrid in a highly scrunched transcription bubble. However, we note that scrunching remains minimal following synthesis of a 3-mer RNA, making it unlikely for stable contact to form between the σ-finger and the RNA–DNA hybrid. Therefore, the subpopulation observed at E* ∼0.72 most likely represents the former scenario where the σ-finger is positioned closer to σR2. This subpopulation disappears upon progression to RP_itc≤4_. Interestingly, for RP_itc≤3_, the total fraction of conformations with disrupted σ-finger contacts to the template DNA reaches ∼42% when considering the combined relative abundances of the E* ∼0.14 (26%) and E* ∼0.72 (16%) subpopulations (Fig. 4C). This value closely matches the observed abundance of displaced σ-finger conformations in RP_itc≤4_ (40%), suggesting that initial disruption of σ-finger-template DNA contacts occurs following synthesis of a 3-mer RNA, while complete displacement of the σ-finger from the active site cleft requires synthesis of a 4-mer RNA.

To determine the status of the transcription bubble for pR during initial transcription, we performed experiments using a pR construct with Cy3 attached to −9 position (pR-[−9-Cy3]) of the template strand. Like results obtained for lacCONS-[+2-Cy3], this construct also shows a rapid decrease in Cy3 fluorescence intensity upon promoter escape [41]. We performed experiments using subsets of NTPs to generate initial transcribing complexes with 2-mer RNA (added ApU), 3-mer RNA (added ApU and GTP), and 4-mer RNA (added ApU, UTP, and GTP). None of these reactions exhibited a fluorescence decay characteristic of a promoter escape reaction (Supplementary Fig. S13A). Together with the fact that it is known escape for pR promoter occurs during RNA extension from a 10- to 11-mer [26], we conclude for pR the σ-finger is displaced well before promoter escape.

Next, we performed real-time experiments with the pR promoter (Fig. 5A). As for the results for lacCONS, we obtained different types of intensity-versus-time trajectories for pR promoter, which could be classified into Class-I and II (42%), Class-III (36%), and Class-IV (22%) molecules. Displacement times obtained from Class-I and Class-II molecules revealed the presence of two sub-populations of molecules exhibiting short (∼17 s, ∼88% of the events) and long (∼185 s, ∼12% of the events) displacement times (Fig. 5B), essentially identical to the results obtained previously for the lacCONS promoter. However, unlike in lacCONS, an analysis of the E* values for the fast and slow displacers before NTP addition revealed similar E* values (∼0.46 and ∼0.48, respectively; Fig. 5C).

### σ-finger displacement during initial transcription: a ribosomal RNA promoter (rrnBP1)

We then analysed σ-finger displacement on a ribosomal RNA promoter (rrnBP1). Such ribosomal RNA promoters are responsible for a large fraction of transcription in bacteria, especially during fast growth; as such, these promoters are characterized by very high turnover rates. In contrast to pR, the rrnBP1 promoter is characterized by a very unstable RP_o_ (lifetime ∼1.45 s) containing a 15-bp unwound bubble and a transcription start site located nine bases downstream of the −10 element; this unusually long spacing (which is 2 nt longer than average) is associated with accumulation of some DNA scrunching in the open complex prior to RNA synthesis initiation [45]. Further, the rrnBP1 promoter is not associated with the production of significant amounts of abortive RNAs [45], and it has been postulated that promoter escape on rrnBP1 may occur as early as RNA lengths of 3 nt [26].

Using the same approaches as for lacCONS and pR promoters, we observed that an RP_o_ formed on an rrnBP1 promoter fragment adopts almost exclusively (94% of molecules) the in-cleft σ-finger conformation, with the rest of the molecules adopting a displaced σ-finger conformation; these results are essentially identical to the profile observed for the lacCONS promoter. In sharp contrast, measurements performed in complexes able to synthesize increasing lengths of RNA showed a large increase in the fraction of displaced conformation as early as RP_itc2_ (29%; Fig. 4D). Strikingly, when dinucleotides used to form RP_itc2_ complexes were washed out from solution—which should lead to dissociation of the RNA dinucleotide and re-formation of the initial RP_o_—the E* histogram still showed a significant fraction of complexes with a displaced σ-finger (24%; Fig. 4E). These results strongly suggest that, on rrnBP1, the formation of the first phosphodiester bond during the synthesis of the dinucleotide provided sufficient interactions to reposition the σ finger in a stably displaced conformation.

To define the point of escape for rrnBP1 and compare it to the point of σ-finger displacement, we used a construct with Cy3 attached to +2 position (rrnBP1-[+2-Cy3]) of the template strand. Like results obtained for lacCONS-[+2-Cy3] and pR-[−9-Cy3], this construct also exhibits a decrease in Cy3 fluorescence upon promoter escape [41]. We performed experiments using subsets of NTPs to generate initial transcribing complexes with 2-mer RNA (added ApU) and 3-mer RNA (added ApU and GTP). None of the two reactions exhibited a fluorescence decay characteristic of a promoter escape reaction (Supplementary Fig. S13B). We thus conclude that promoter escape in rrnBP1 does not occur upon formation of a 3-mer RNA. Taken together, our results for the rrnBP1 promoter indicate that the σ-finger is displaced before promoter escape and as early as the synthesis of a 2-nt RNA.

Real-time experiments with rrnBP1 promoter also showed different types of intensity-versus-time trajectories, which could be classified into Class-I and II (39%), Class-III (23%), and Class-IV (38%) molecules. Further analysis of Class-I and Class-II molecules revealed two subpopulations with displacement events of ∼3.8 and ∼36 s, and E* efficiencies of ∼0.49 before NTP addition (Fig. 5D and E). The subpopulation exhibiting very fast displacement events was similar in abundance to subpopulations exhibiting fast displacement kinetics for the other two promoters (84% for rrnBP1 vs 88% for lacCONS and pR) but exhibited much faster displacement times (∼3.8 s for rrnBP1 versus ∼14 s for lacCONS and ∼17 s for pR). This striking difference is a likely consequence of the distinctly different nature of RPo formed at the rrnBP1 promoter and its very early point of displacement (after synthesis of 2-nt RNA). The slow-displacing subpopulation on rrnBP1 is also five-fold faster compared to the slow-displacing population in lacCONS (∼188 s) and pR (∼185 s) (Figs 3C and 5B).

Overall, the results of the real-time experiments reveal subpopulations of molecules showing very slow displacement events of ∼180–200 s for lacCONS and pR. Given the distinctly different point of displacement of the σ-finger for lacCONS (very late displacement) and for pR (early displacement), we suggest that the majority of the displacement time is spent before initial transcription begins. A likely explanation is that the slow displacers represent RPo molecules residing in a transcriptionally inactive conformation for a substantial duration. Similar functional heterogeneity among RPo molecules has been recently reported in studies employing the pR promoter [46] and the lacCONS promoter [47]. A subpopulation with such markedly slow displacement (∼180 s) is absent on rrnBP1. However, since we observe a very early displacement (after 2-nt RNA synthesis) for rrnBP1, it is unlikely the subpopulation with ∼36 s displacement time spent most of this time in initial transcription. Therefore, we suggest that, similar to the other two promoters, a long-lived transcriptionally inactive RP_o_ state also occurs on rrnBP1.

## Discussion

### The σ-finger is displaced from an in-cleft conformational state during initial transcription

In this study, we developed a new FRET-based approach for detecting σ-finger conformations in single transcription complexes and captured the σ-finger conformational profile throughout initial transcription (from RP_o_ complexes to early elongation complexes) for three promoters with different transcription initiation profiles.

Notably, our construct reports on the position of the base of the σ-finger (via labelling at residue 511) and thus does not capture motions restricted to the tip of the finger (residues 513–519), such as the motions observed in a structural study examining analogues of initial transcribing complexes with increasing RNA lengths [18] (Supplementary Fig. S1B). Therefore, the large FRET changes observed in our assays with different promoter fragments report on full displacement of the σ-finger from ‘in-cleft’ conformations in early stages of transcription initiation. The observed FRET decrease upon RNA synthesis suggests that the σ-finger moves out of the RNAP main cleft and away from the leading edge of RNAP (where the other FRET probe is attached), likely moving towards the dsDNA upstream of the transcription bubble. Such displaced conformations have been proposed but were never directly observed in structural studies, likely due to high mobility of this displaced structural module.

**Figure 6.**
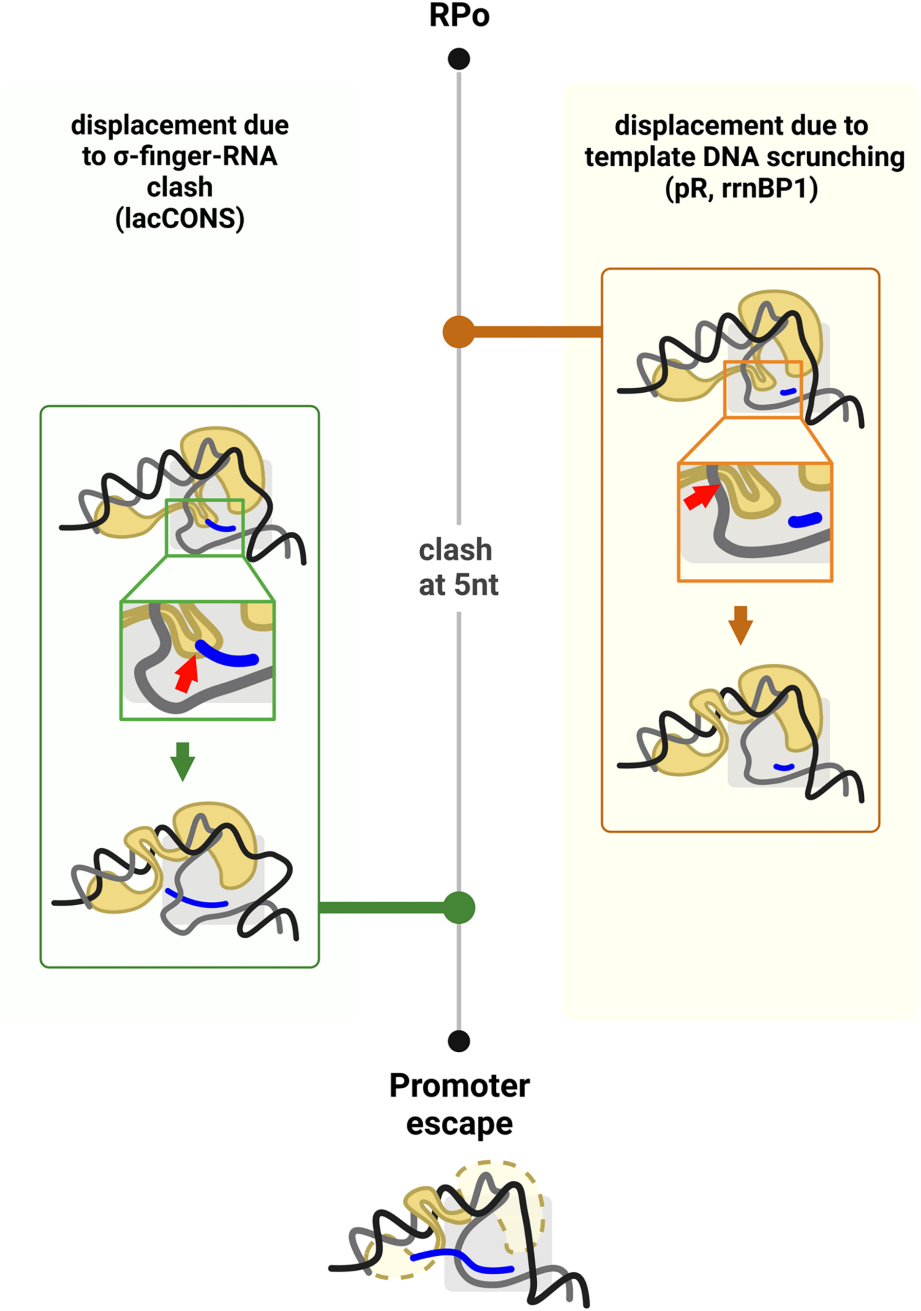
Two distinct, promoter-dependent mechanisms for the displacement of the σ-finger during initial transcription. Left, displacement occurs due to the clash of the 5′-end of the RNA with the σ-finger. Right, displacement occurs due to altered interactions of the finger with template DNA driven (directly or indirectly) by DNA scrunching. Created in BioRender. Mazumder, A. (2025) https://BioRender.com/22tuag8.

Importantly, for all promoters studied, σ-finger displacement—and thus disruption of σ-finger–template DNA contacts—occurs at RNA lengths shorter than those required for the final step of promoter escape, which releases RNAP from the promoter and marks the end of initial transcription. Specifically, σ-finger displacement was observed after synthesizing 10-nt, 3-nt, and 2-nt RNAs for the *lacCONS*, *pR*, and *rrnBP1* promoters, respectively, whereas promoter escape occurred at later stages (based on results in Supplementary Figs S7 and S13). Our findings demonstrate that σ-finger displacement precedes the final step of promoter escape, supporting a multi-step escape model in which σ-DNA interactions (with both ssDNA and dsDNA) are disrupted sequentially (Fig. 6). This aligns with recent biochemical evidence from ensemble studies of initial transcription using pR promoter derivatives [46].

### σ-finger displacement can occur via different mechanisms depending on the promoter

Structural work on the σ^70^ finger using synthetic transcription initiation scaffolds containing different lengths of RNA revealed that, as the RNA length increases, the 5′-end of the RNA clashes with the σ-finger, and for sufficiently long RNAs, the tip of the σ-finger gets displaced, folding back on itself in a manner analogous to compression of a spring, with each stepwise increase in RNA length leading to greater displacement of the tip of the finger [18]. Very recent structural work on initial transcribing complexes formed by σ^54^ RNAP and a synthetic promoter scaffold containing increasing lengths of pre-synthesized RNA showed that the ‘RII-finger’, a σ^54^ element functionally equivalent to the σ^70^ finger, also folds back on itself when the RNA reaches ∼7 nt, at which point a steric clash between the RNA and the RII-finger occurs in a manner analogous to the σ^70^ finger [48]. These studies support the hypothesis that full displacement of the σ-finger away from the path of the extending RNA follows an obligatory steric clash with the extending RNA. As a corollary of this ‘obligatory steric clash’ hypothesis, the exact point of displacement for different promoters should be dictated by the extent to which the σ-finger protrudes inside the unwound transcription bubble and the strength of the contacts it makes with the template single-stranded DNA (ssDNA) and the RNA.

Consistent with the obligatory steric clash hypothesis, σ-finger displacement from the cleft occurred late on the lacCONS promoter—after synthesis of 10-nt RNA (Fig. 6). Intriguingly, however, and contrary to the obligatory steric clash hypothesis, our measurements with the pR and rrnBP1 promoters showed that displacement occurred at RNA lengths of only 3 nt and 2 nt, respectively, lengths much shorter than the 5-nt RNA length expected for a steric clash of the 5′-end of the RNA with the σ-finger for these promoters (Fig. 4C and D). We thus suggest that, even before the ensuing steric clash between the σ-finger and the nascent RNA in the active-site cleft of RNAP, a steric clash between the σ-finger and the scrunched template DNA, and/or a weakening of interactions between the σ-finger and the template ssDNA due to scrunching, triggers disruption of the σ-finger contacts, enabling full displacement of the σ-finger (Fig. 6). Notably, the σ-finger interacts with template bases −3/−4 with promoters of a canonical discriminator of 6 nt (such as lacCONS and λPr [7]) and template bases −4/−5 with promoters with a longer discriminator (such as *rrn*BP1^43^); the template bases involved in these contacts are different in all 3 promoters, raising the possibility of a DNA-sequence determinant for finger displacement. Further, the recent work on the σ^54^ RII-finger displacement also showed altered interactions between the RII-finger and the template DNA due to DNA scrunching [48].

### Influence of the different mechanisms of σ-finger displacement on the ‘initiation pause’

Previous single-molecule and crosslinking studies of initial transcription with the lacCONS promoter identified an ‘initiation pause’ during extension of a 6-nt to a 7-nt RNA; the same study showed that deletion of the σ-finger resulted in significantly reduced pause lifetimes as RNA extends from 6-nt to 7-nt, establishing the σ-finger as a crucial determinant of pausing during initial transcription, in addition to the initial transcribed sequence [23, 42, 43]. We suggest that, for lacCONS, the σ-finger clashes with the RNA after synthesis of a 5-nt RNA, while making additional stabilizing contacts with the 5′-end of the RNA, as observed in [18], likely raising the energy barrier of translocation during initial transcription and enhancing pausing, as observed in [23, 49, 50]. Further extension of RNA beyond the 7-mer results in additional scrunched DNA and additional folding of the tip of the σ-finger, as observed in [18]—both structural elements are capable of acting like a compressed spring and storing energy, which can be released to disrupt RNAP promoter contacts during late stages of initial transcription, eventually enabling escape from the promoter. Our observation of the σ-finger getting displaced at 10-nt RNA length—well before the point of promoter escape for lacCONS (Supplementary Fig. S7)—suggests the stored energy in the folded σ-finger may indeed be used up in this step, disrupting the contacts formed between the σ-finger and the RNA, facilitating its displacement from the active centre cleft, and clearing the path for promoter escape.

However, for the pR and rrnBP1 promoters, an early displacement of the σ-finger well before its expected clash with the nascent RNA chain indicates that the additional translocation energy barrier posed by the σ-finger (as e.g. in the case of lacCONS) would be absent. Hence, one expects significantly reduced lifetimes of the ‘initiation pause’ for these promoters, leading to much shorter times spent in initial transcription—a hypothesis that warrants future high-throughput studies linking pausing times with a large library of promoter sequences based on methods developed in [51]. We thus suggest that the σ-finger and its displacement can act as a major determinant of rates of transcription initiation and promoter escape.

### Interactions with the 5′-end of nascent RNA modulate σ-finger displacement

In contrast to our results obtained for primer-dependent transcription initiation, primer-independent transcription initiation yielded strikingly different results for the lacCONS promoter, with σ-finger displacements observed at a much earlier stage (after synthesis of 5-nt RNA instead of 10-nt RNA). Primer-dependent transcription involves synthesis of an RNA with a hydroxyl group at the 5′-end (5′-OH), while primer-independent transcription involves an RNA with a triphosphate at the 5′-end (5′-ppp). Since the bacterial σ-finger is negatively charged under physiological conditions (due to the presence of a stretch of four acidic residues in the σ-finger: D513, D514, E515, and D516), our results indicate that the bulky, negatively charged triphosphate group at the 5′-end of the growing RNA repels the negatively charged σ-finger, triggering σ-finger displacement at RNA lengths significantly shorter than that observed for reactions involving 5′-OH ends of the RNA. As argued in the previous section, a displacement after 5-nt RNA synthesis would result in a lower energy barrier of translocation for the initiation pause (observed at +6) for lacCONS, and consequently one expects a much shorter pause lifetime in case of primer-independent initial transcription for this promoter. In agreement with this result, Dulin *et al.* [32] previously reported a ∼2.5-fold decrease in pause duration for primer-independent initiation as compared to primer-dependent initiation at the same promoter.

Notably, most transcription under exponential growth involves primer-independent initiation, while primer-dependent initiation becomes prevalent during stationary phase [52, 53]. Our results indicate that along with the variability in transcription start site selection [53], σ-finger interactions with the 5′-end of the RNA may be used by bacteria to tune gene expression in a growth-dependent fashion, since an early σ-finger displacement in primer-independent initiation could facilitate promoter escape and result in higher transcription output during exponential growth, while larger timescales for σ-finger displacement in primer-dependent initiation, as shown by real-time measurements, could lead to reduced transcription during stationary phase. We further note that transcription with a 5′ non-canonical initiating nucleotide like NAD+ (Nicotinamide Adenine Dinucleotide) [54], which contains a significantly bulkier group at the 5′ end, would also likely involve altered σ-finger displacement profile.

### σ-finger displacement on a single promoter occurs at substantially different timescales

Our real-time measurements of displacement time for primer-dependent initiation on lacCONS revealed that only ∼25% of the molecules show displacement. Since we miss the displacement event in ∼20% of the molecules (due to probe photobleaching prematurely terminating our FRET time trajectories), our results imply that ∼55% of the molecules are either transcriptionally inactive or remain in early stages of initial transcription where the σ-finger is still not displaced. These results are consistent with previous reports from biochemical and single-molecule experiments showing the presence of a class of transcription complexes that remain in abortive initiation and do not proceed to elongation [32, 44, 55–58].

The molecules showing displacement belonged to two subclasses, exhibiting either a short (5–20 s) or much longer (>150 s) displacement time for the lacCONS promoter. These times are strikingly like the promoter-escape times [∼13 s (96%) and ∼200 s (4%)] observed in a single-molecule study on the same promoter [47]. Further analysis revealed σ-finger conformations have different E* distributions in the initial state (i.e. in the RPo state) for the kinetically different molecules. This indicates that different conformational ensembles result in the observed heterogeneity in displacement times, i.e. conformational ensembles with slightly lower E* values result in faster displacement times, indicating the positioning of the σ-finger inside the active site cleft to be an important determinant in the kinetics of initial transcription. How do molecules with different σ-finger positions result in different displacement kinetics? We envision that the basis for this resides in altered pre-organization of the template strand by the differently positioned σ-fingers and/or in differences in the ensuing steric clash between the nascent RNA and the differently positioned σ-finger modules.

In contrast, our analysis on the pR promoter (which showed displacement early in initial transcription, after synthesis of a 3-mer RNA) showed both short and long displacement times, as in the lacCONS case; however, both short and long displacement times show σ-finger conformations with similar E* values before NTP addition. What gives rise to this kinetic heterogeneity? Given the short RNA length needed for σ-finger displacement on the pR promoter, a steric clash with the RNA during initial transcription can be ruled out; further, our assay could not pick up significantly different σ-finger conformations in the kinetically distinct subpopulations, and, as such, our results on λP_R_ do not support the presence of an altered pre-organization of the template strand driven by different σ-finger conformations.

Instead, we speculate that the kinetic heterogeneity in this case arises from conformational heterogeneity in RP_o_ molecules, which involve different template strand organizations as observed in a recent study on RP_o_ formed on a pR promoter [19]; in that study, the authors report one RP_o_ conformation with template strand conformations incompatible with binding initiating nucleotides, and hence incompatible with transcription initiation [19]. In this scenario, the long σ-finger displacement times for a subpopulation of transcription complexes on the pR promoter could originate from the rate-limiting step of the conversion of the inactive conformation of RP_o_ to the active conformation.

Our analysis of the rrnBP1 promoter also showed kinetically heterogeneous σ-finger displacement, with both fast-displacers and slow-displacers showing displacement much faster than the other two promoters. This heterogeneity is intriguing, since the displacement times for rrnBP1 under our conditions essentially report on the binding of the dinucleotide primer (and not on rate of RNA synthesis); in other words, primer binding alone can trigger σ-finger displacement. The observed heterogeneity again indicates the presence of distinct RP_o_ conformational states (as for the pR promoter), with different template strand organizations, which in turn result in distinct binding efficiency of the dinucleotide primer to the RNAP active site. Similar to the pR promoter, we do not observe significant differences in E* values for the σ-finger conformation for the kinetically different molecules.

Taken together, our study identifies displacement of the σ-finger as a key step that influences the kinetics of initial transcription and reveals different mechanisms that influence this process; these mechanisms can be leveraged by the bacteria to regulate transcription at many levels (different genes, different sets of genes, or different physiological states). Such conformational control may also offer a new target for generating antibiotics, e.g. by identifying small molecules that severely delay or block promoter escape on pivotal genes, such as the rRNA genes.

Importantly, we note that the σ-finger displacement register, the kinetics of displacement, and likely the underlying displacement mechanism vary greatly depending on the promoter being studied. However, the precise sequence determinants responsible for these differences remain unclear, and a systematic investigation of the σ-finger displacement mechanism across a broad range of promoter sequences—with randomized sequences for the template DNA strand positions that contact the σ-finger, as well as other critical promoter elements like the −10, −35, and initial transcribed sequence—could provide a comprehensive answer. To address this, we are developing a high-throughput single-molecule experiment for mapping of σ-finger conformational changes combined with *in situ* single-molecule sequencing of a large library of DNA sequences in a future study based on technologies already developed in [51].

We note that structural modules similar to the bacterial σ-finger (in that they protrude into the cleft of transcription initiation complexes, interact with template ssDNA, and occupy the path of the nascent RNA chain) are present in other kingdoms of life [e.g. TFB zinc ribbon and CSB of the archaeal RNAP [10]; Rrn7 zinc ribbon and B reader of RNAP-I [14, 15]; TFIIB zinc ribbon and B reader of RNAP-II [11, 13, 59, 60]; and Brf1 zinc ribbon of RNAP-III [16, 17]. We suggest these modules likely behave in a manner analogous to the bacterial σ-finger and influence the kinetics of transcription and gene regulation in both archaea and higher eukaryotes in an analogous manner.

## Supplementary Material

gkaf857_Supplemental_File

## Data Availability

All our time-trace data are available to anyone upon request. MATLAB software package TwoTone is available at https://doi.org/10.5281/zenodo.16758905.
