# Supplementary material for: The displacement of the σ70 finger in initial transcription is highly heterogeneous and promoter-dependent
Source: Nucleic Acids Res. 2025 Sep 12;53(17):gkaf857. doi: 10.1093/nar/gkaf857 (PMC12445709; doi:10.1093/nar/gkaf857)
Supplement: gkaf857_Supplemental_File [file gkaf857_supplemental_file.docx]

**SUPPORTING INFORMATION FOR**

**The displacement of the σ^70^ finger in initial transcription is highly heterogeneous and promoter-dependent**

Anna Wang^a,b,✝^, Andrew G. Fletcher^d^, Pratip Mukherjee^c^, David C. Grainger^d^, Abhishek Mazumder^a,b,c,✝*^, and Achillefs N. Kapanidis^a,b,*^

^a^ Biological Physics Research Group, Department of Physics, University of Oxford, Oxford OX1 3PU, United Kingdom

^b^ Kavli Institute for Nanoscience Discovery, Dorothy Crowfoot Hodgkin Building, University of Oxford, Sherrington Road, Oxford, OX1 3QU, United Kingdom

^c^ Structural Biology and Bioinformatics Division, CSIR-Indian Institute of Chemical Biology, 4 Raja S. C. Mullick Road, Jadavpur, Kolkata-700032, India

^d^ School of Biosciences, University of Birmingham, Edgbaston, Birmingham B15 2TT, UK

*To whom correspondence should be addressed: [kapanidis@physics.ox.ac.uk](mailto:achillefs.kapanidis@physics.ox.ac.uk); [abhishek@iicb.res.in](mailto:abhishek@iicb.res.in)

**This PDF file includes:**

Table S1

Figures S1 to S14

SI References

**Table S1.** DNA sequences.

| **lacCONS+12C** construct for measurements in Figs. 1D, 2A (RP_itc2_, RP_itc7_, RP_itc11_), 2B (RP_itc2_, RP_itc11_), S5, S6, S12 | |
| --- | --- |
| Non-template strand | 5’‑(biotin)AGGCTTGACACTTTATGCTTCGGCTCGTATAATGTGTGGAATTGTGAGAGCGGATAACAATTTC-3’ |
| Template strand | 5‑ GAAATTGTTATCCGCTCTCACAATTCCACACATTATACGAGCCGAAGCATAAAGTGTCAAGCCT-3’ |
| **lacCONS+8C** construct for measurements in Fig. 2B (RP_itc7_) | |
| Non-template strand | 5’‑(biotin)AGGCTTGACACTTTATGCTTCGGCTCGTATAATGTGTGGAATTGTGCGAGCGGATAACAATTTC-3’ |
| Template strand | 5’‑GAAATTGTTATCCGCTCGCACAATTCCACACATTATACGAGCCGAAGCATAAAGTGTCAAGCCT-3’ |
| **lacCONS+15C** construct for measurements in Figs. 2A (RD_e14_), 2B (RD_e14_), 3, S8, S9, S10 | |
| Non-template strand | 5’‑(biotin)AGGCTTGACACTTTATGCTTCGGCTCGTATAATGTGTGGAATTGTGAGGAGGACGGATAACAATTTC-3’ |
| Template strand | 5’‑ GAAATTGTTATCCGTCCTCCTCACAATTCCACACATTATACGAGCCGAAGCATAAAGTGTCAAGCCT-3’ |
| **rrnB P1** construct for measurements in Figs. 4B, 4C, 5D-F | |
| Non-template strand | 5’‑(biotin)CTCTTGTCAGGCCGGAATAACTCCCTATAATGCGCCACCACTGACACGGAACAACGGCAAACAC-3’ |
| Template strand | 5’‑ GTGTTTGCCGTTGTTCCGTGTCAGTGGTGGCGCATTATA GGGAGTTATTCCGGCCTGACAAGAG-3’ |
| **pR** construct for measurements in Figs. 4A, 5A-C | |
| Non-template strand | 5’‑(biotin)ATCTATCACCGCAAGGGATAAATATCTAACACCGTGCGTGTTGACTATTTTACCTCTGGCGGTGATAATGGTTGCATGTAGTAAGGAGGTGGTATGGAAT-3’ |
| Template strand | 5’‑ ATTCCATACCACCTCCTTACTACATGCAACCATTATCACCGCCAGAGGTAAAATAGTCAACACGCACGGTGTTAGATATTTATCCCTTGCGGTGATAGAT-3’ |

**
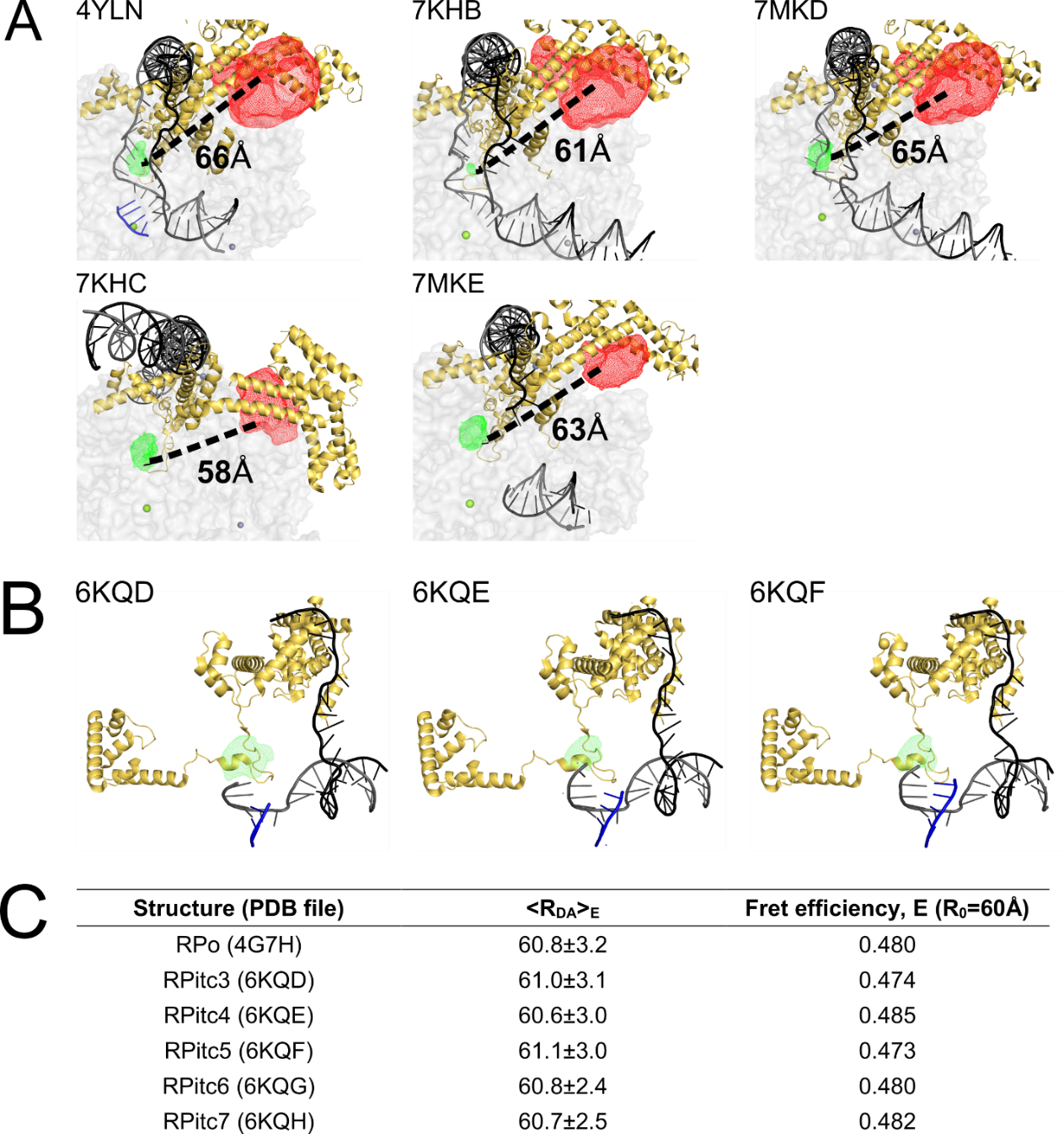
**

**Fig. S1.** Accessible volume measurements for labelling the base of the σ-finger.

(A) Accessible volume clouds and measurements between σ^70^ residue 511 an σ^70^ residue 366 for PDB structures 4YLN, 7KHB, 7MKD, 7MKE, 7KHC.

(B) Accessible volume modelling of fluorescent probe Cy3B placed at position σ^70^ residue 511 (σ^A^ residue 321) on complexes with 3 (6KQD), 4 (6KQE) and 5 (6KQF) nucleotides of RNA. σ^70^ is straw coloured; accessible volume modelling shown is green; RNA is in blue; template DNA in gray, and non-template DNA in black.

(C) Accessible volume distance measurements between a label at the base of the σ-finger (σ^A^ residue 319) and σ^70^ residue 366 (σ^A^ residue 174) showing that a label base of the σ-finger is not sensitive to movements at the tip of the σ-finger. σ^A^ residue 319 was used for these comparisons as σ^A^ residue 321 was not present in PDB files 6KQG and 6KQH.

**
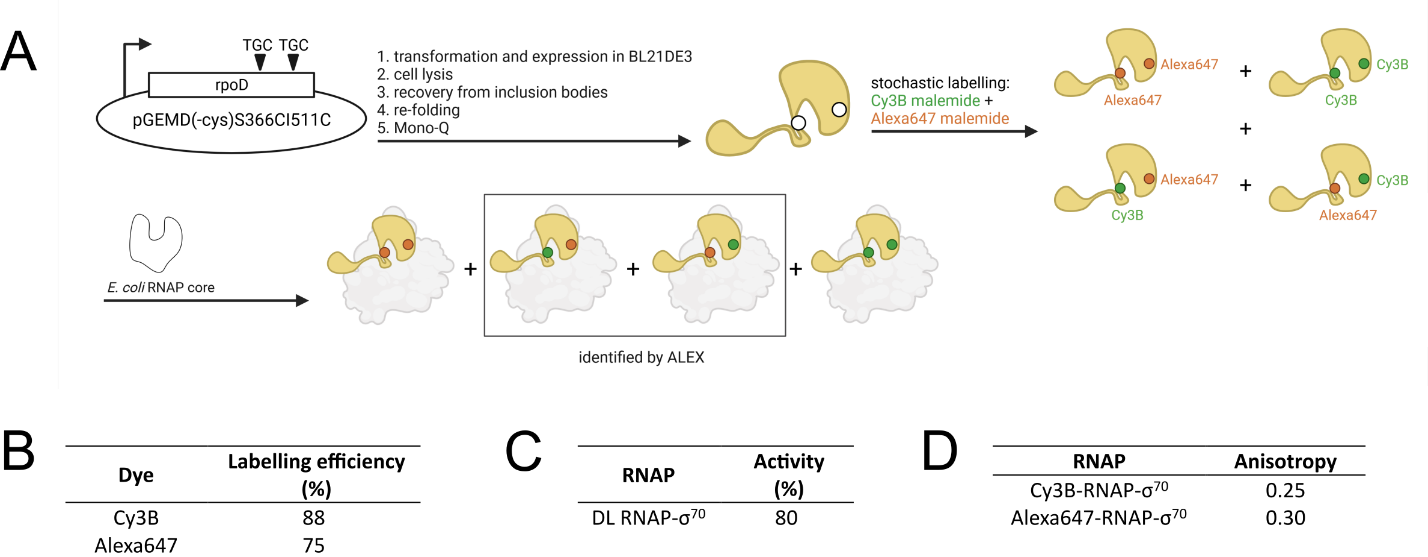
**

**Fig. S2.** Labelling of the RNAP-σ^70^ construct at positions 511 and 366 with dyes Cy3B and Alexa647.

(A) Summary of the labelling method.

(B) Dye labelling efficiencies.

(C) Transcriptional activity of the DL RNAP-σ^70^ construct.

(D) Anisotropies of fluorescent dyes measured on RNAP-σ^70^


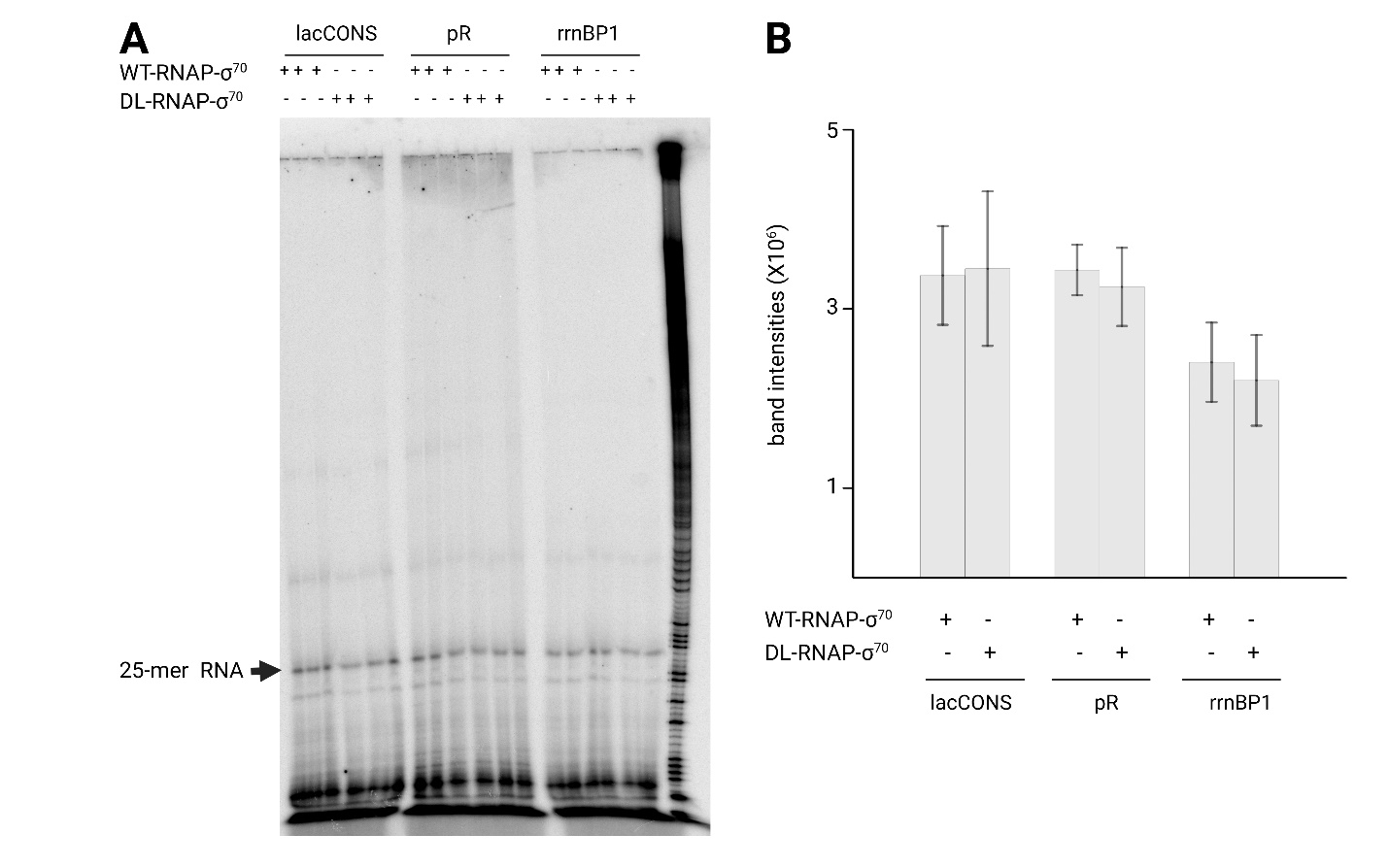


**C**

**
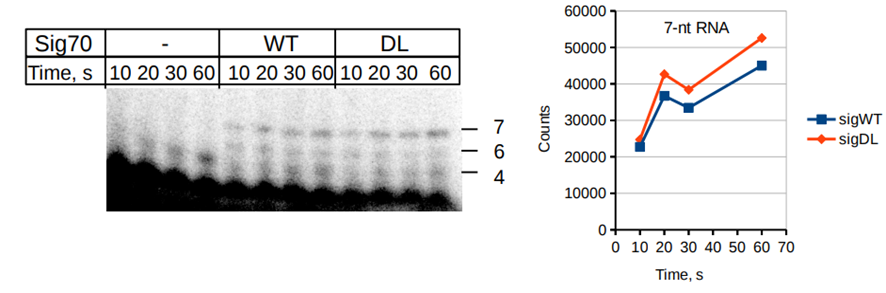
**

**Fig. S3.** Functional assays of double labelled σ^70^ derivative.

1. *In vitro* transcription assay comparing initial transcription profiles obtained using the DL RNAP-σ^70^ and a wild type RNAP-σ^70^ on all three promoters (lacCONS; pR; and rrnBP1; sequences in Table S1).
2. Extracted band intensities of the 25-nt run-off RNA product formed in the in-vitro transcription assay.
3. In-vitro transcription assay showing relative amounts of 6-mer and 7-mer RNA produced during initial transcription using subset of NTPs directing transcription up to a 7nt long RNA for the lacCONS promoter (sequence as in Table S1).

**
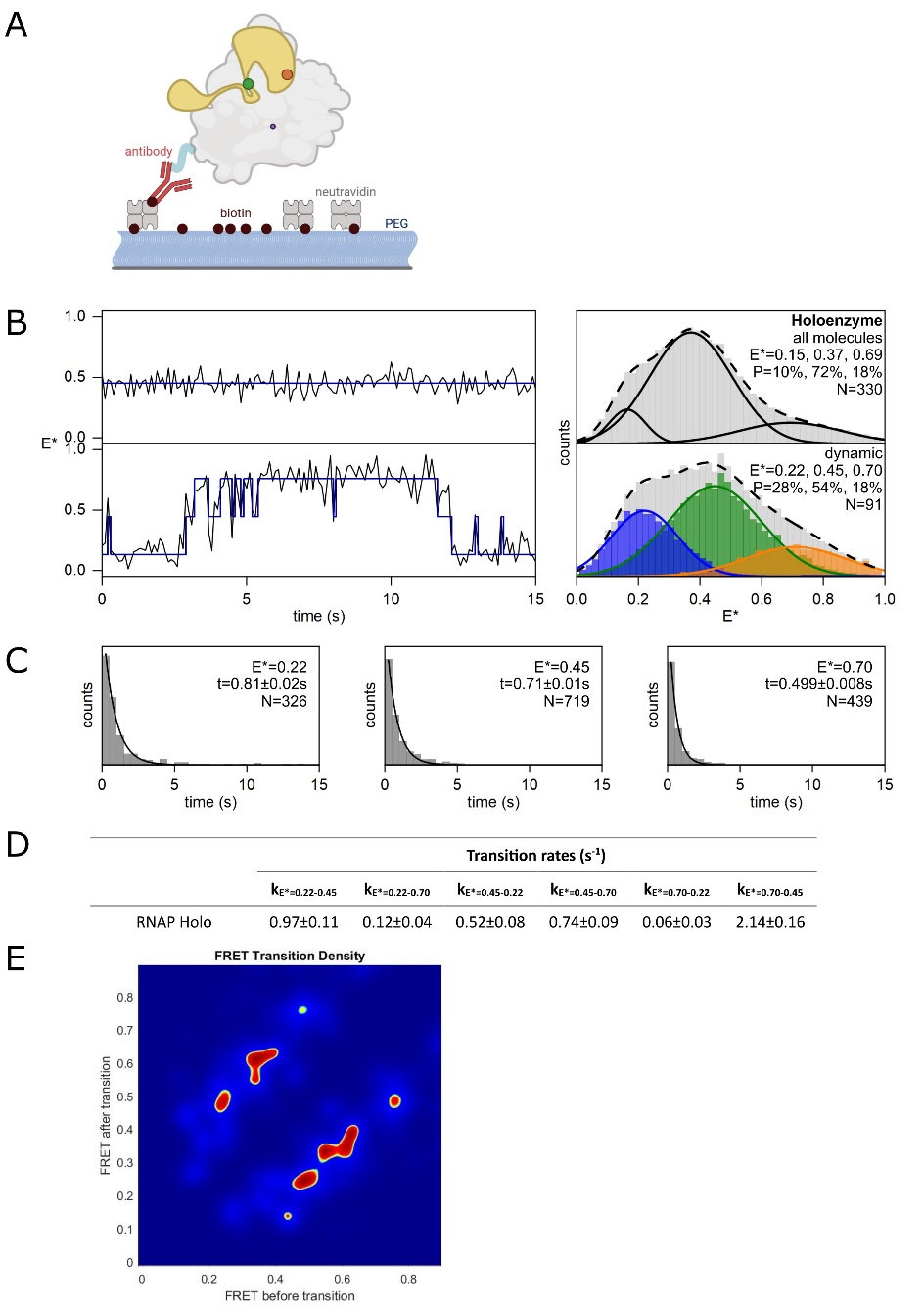
**

**Fig. S4.** *In vitro* smFRET results with DL RNAP-σ^70^.

(A) DL RNAP-σ^70^ was immobilized via a hexahistidine tag on a glass slide functionalized with anti-hexahistidine tag antibody.

(B) smFRET data for the σ-finger in DL RNAP Holoenzyme complexes showing static (*upper*) and dynamic (*lower*) behaviour. Left, representative traces of static and dynamic behaviour. Right, E* histograms formed as a result of hidden Markov modelling, and Gaussian fitting of sub-populations.

(C) Dwell time histograms of each of the states found by hidden Markov modelling of traces exhibiting dynamic behaviour.

(D) Transition rates found by hidden Markov modelling of traces exhibiting dynamic behavior.

(E) Transition density plot showing the frequency of transition between the conformational subpopulations.

**
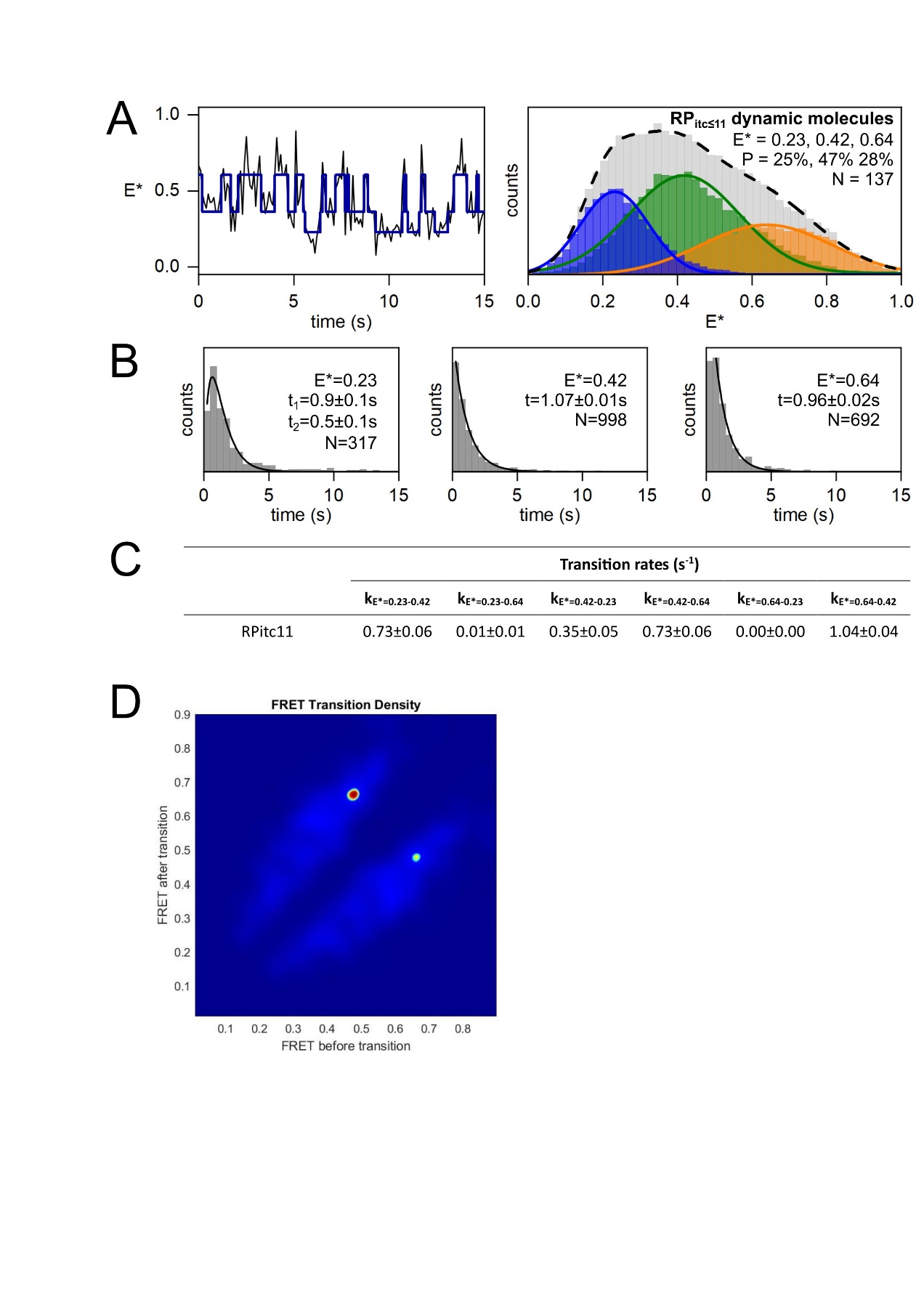
**

**Fig. S5.** *In vitro* smFRET data for dynamic σ-finger RP_itc<11_ molecules formed with lacCONS promoter and ApA initiating dinucleotide.

(A) Left, representative traces of dynamic behaviour. Right, E* histograms formed as a result of hidden Markov modelling, and Gaussian fitting of sub-populations.

(B) Dwell time histograms of each of the states found by hidden Markov modelling of traces exhibiting dynamic behaviour.

(C) Transition rates found by hidden Markov modelling of traces exhibiting dynamic behaviour.

(D) Transition density plot for RP_itc<11_ showing the frequency of transition between the conformational subpopulations.

**
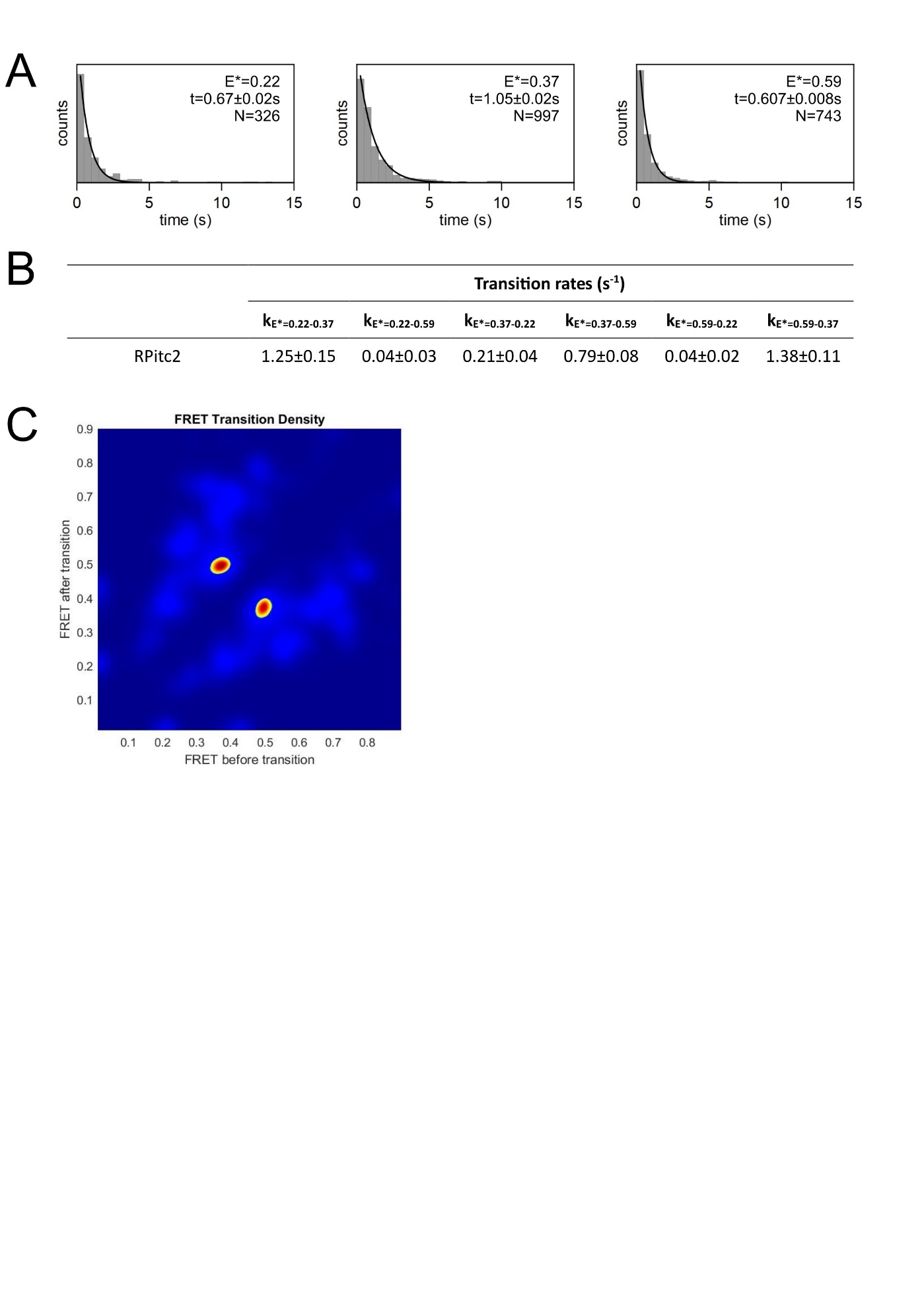
**

**Fig. S6.** Dwell time histograms and transition rates for RP_itc2_ molecules formed with the lacCONS promoter and ApA initiating dinucleotide. States are found by hidden Markov modeling of individual smFRET trajectories exhibit conformational dynamics of the σ-finger.

(A) Dwell time histograms of each of the states found by hidden Markov modelling of traces exhibiting dynamic behaviour.

(B) Transition rates found by hidden Markov modelling of traces exhibiting dynamic behaviour.

(C) Transition density plot for RP_itc2_ showing the frequency of transition between the conformational subpopulations.

**
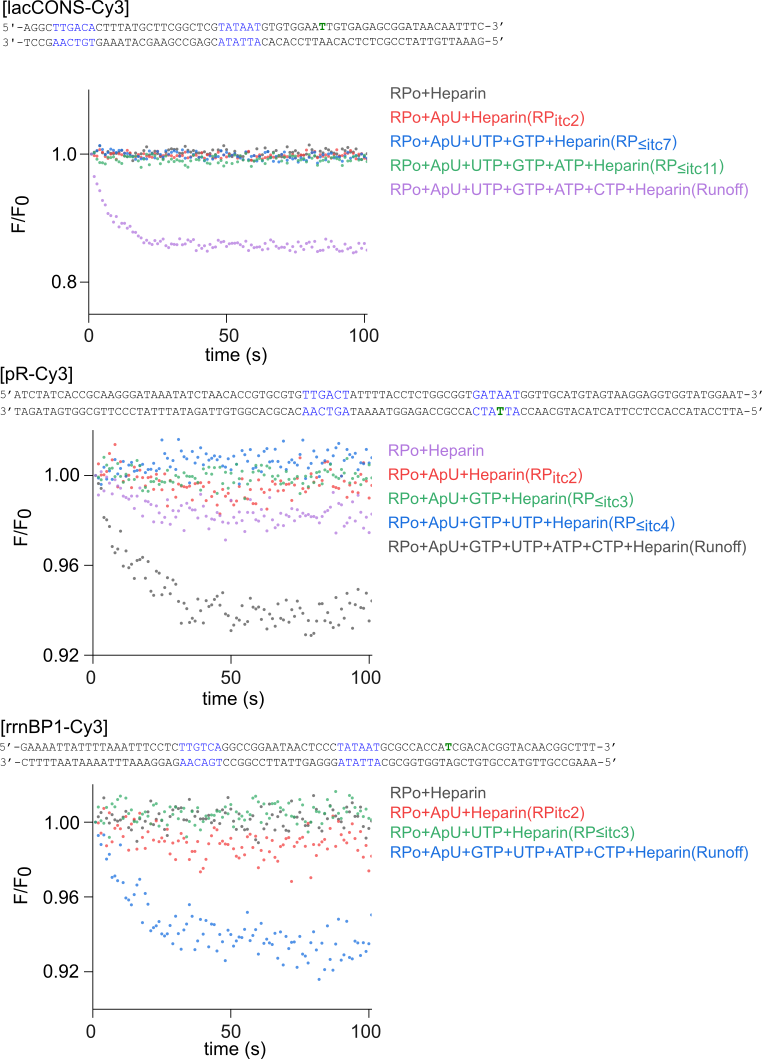
**

**lacCONS-[+2-Cy3]**

**Fig. S7. Promoter escape assay for lacCONS (top)** Sequence of lacCONS-[+2-Cy3] construct used for the promoter escape assay. (bottom) fluorescence intensity decays corresponding to the addition of different subsets of NTPs.

**
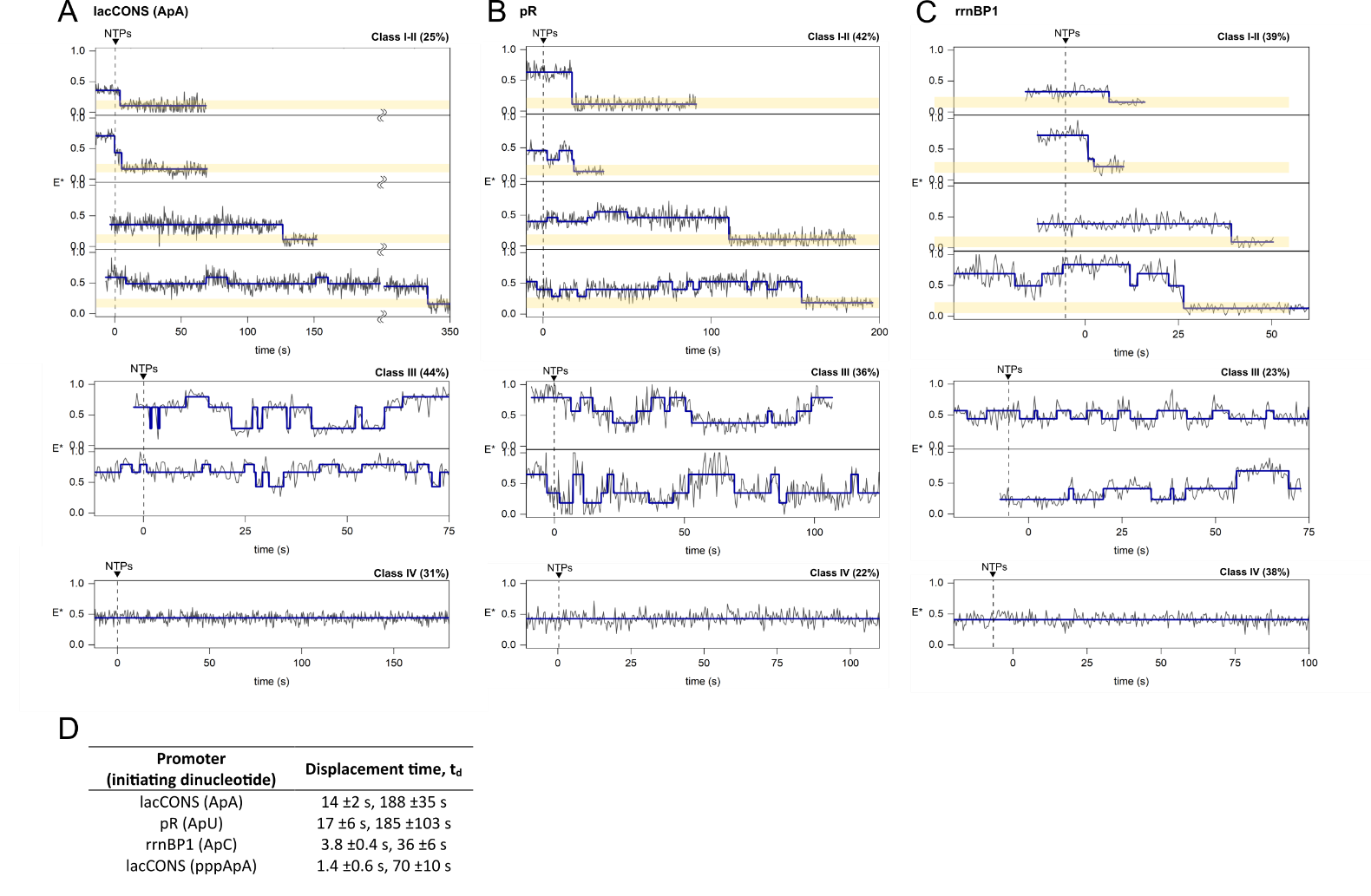
**

**Fig. S8.** Example traces of each class of E* time trajectories obtained in real-time σ-finger experiments involving the lacCONS, pR and rrnBP1 promoters, and 5’-OH initiating dinucleotide:

(A) lacCONS promoter.

(B) pR promoter.

(C) rrnBP1 promoter.

(D) Displacement times.


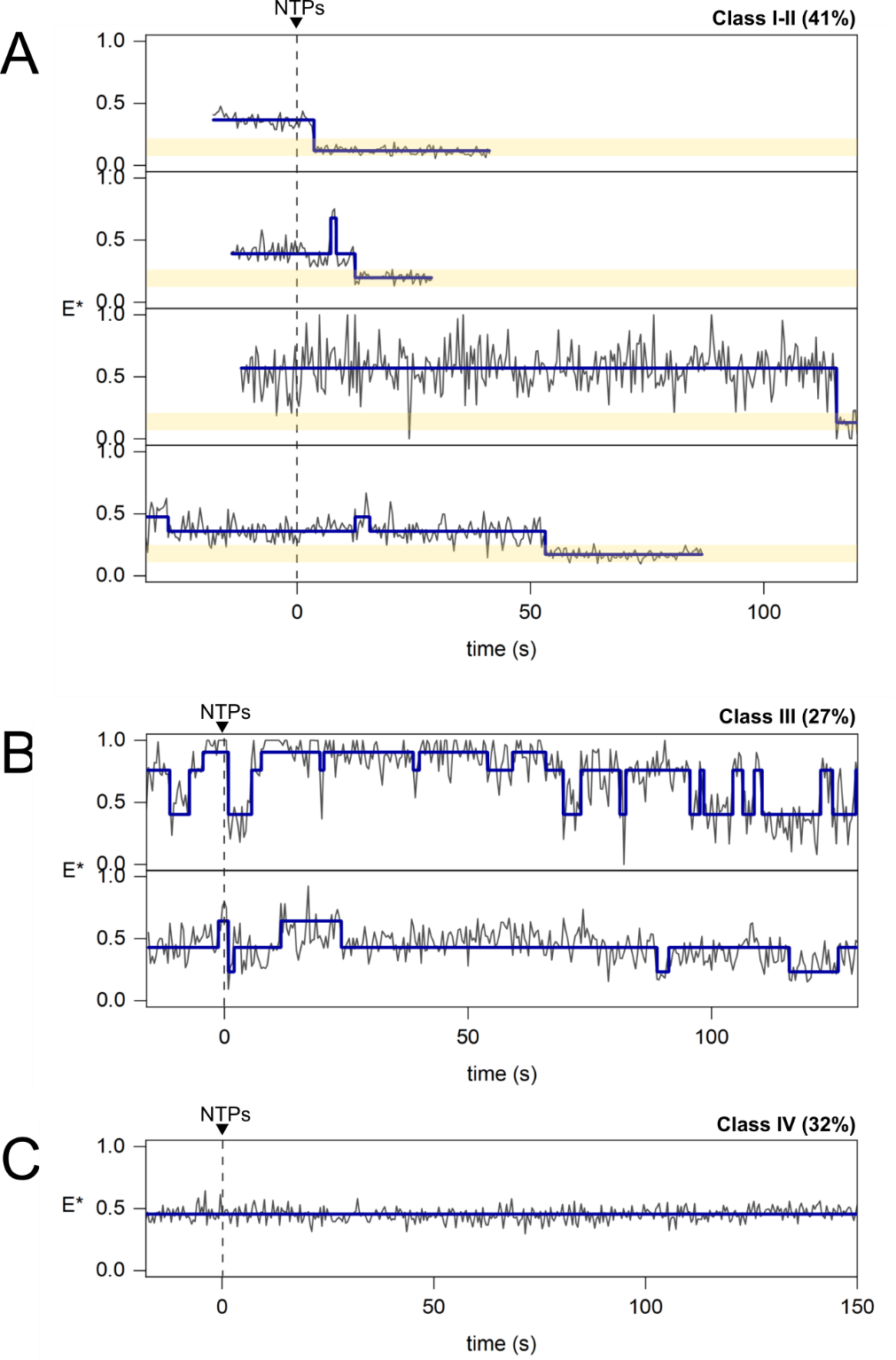


**Fig. S9.** Example traces of each class of E*-time trajectories obtained in real-time σ-finger experiments involving the lacCONS promoter and pppApA initiating dinucleotide.

(A) Class I and Class II. The conformation after displacement is highlighted in yellow.

(B) Class III.

(C) Class IV.


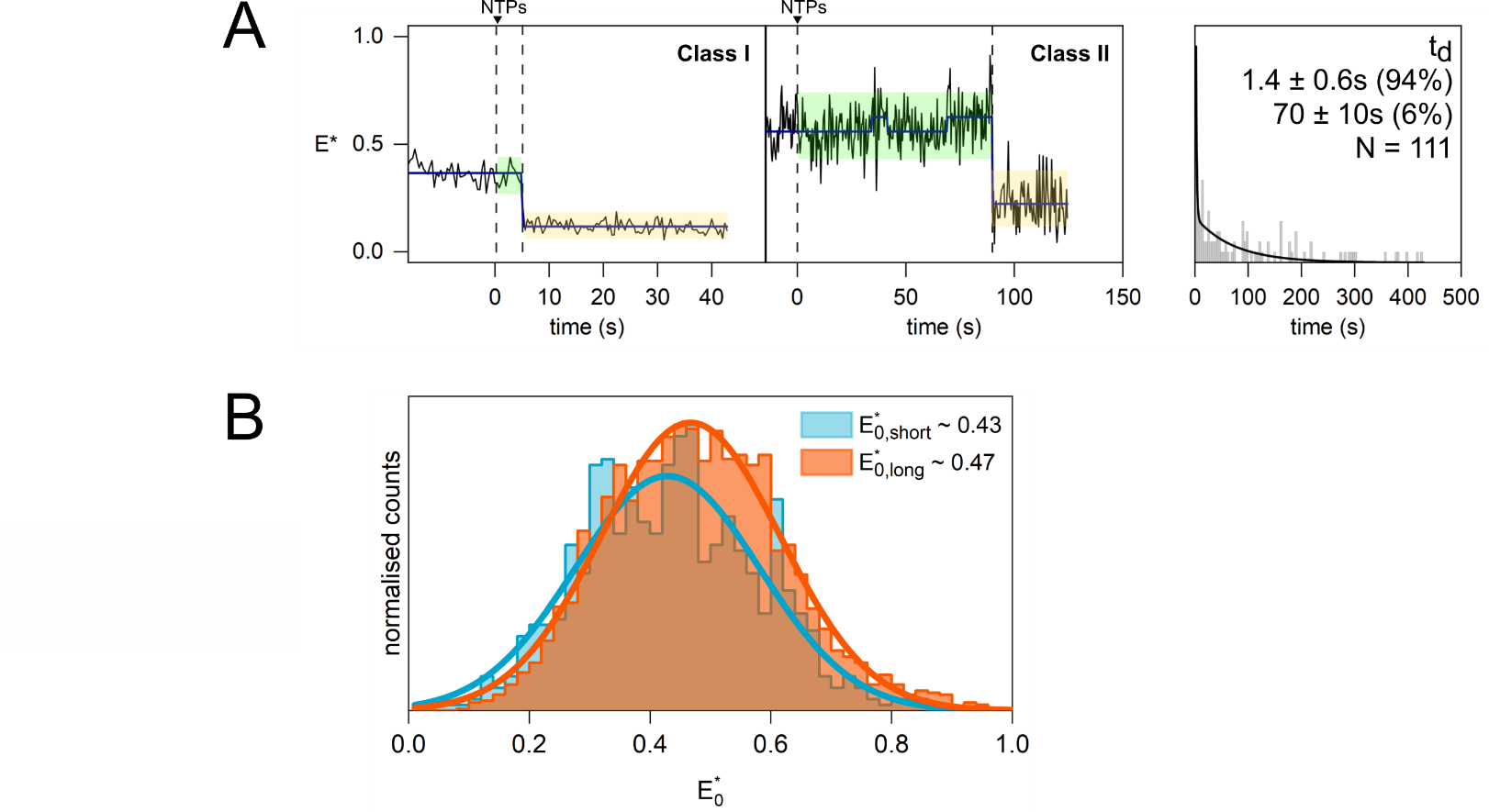


**Fig. S10.** smFRET data tracking the σ-finger for the lacCONS promoter and synthesis of RNA with a 5’-triphosphate end.

(A) Representative E*-time trajectories and dwell time histogram for the time to displacement, t_d_, fitted to a double-exponential decay (black line). The conformation between NTP addition and displacement is highlighted in green, and the conformation after displacement is highlighted in yellow.

(B) Histograms showing the σ-finger conformation before NTP addition of Class-I (E*_0,short_; blue) and Class-II (E*_0,long_; red) molecules.


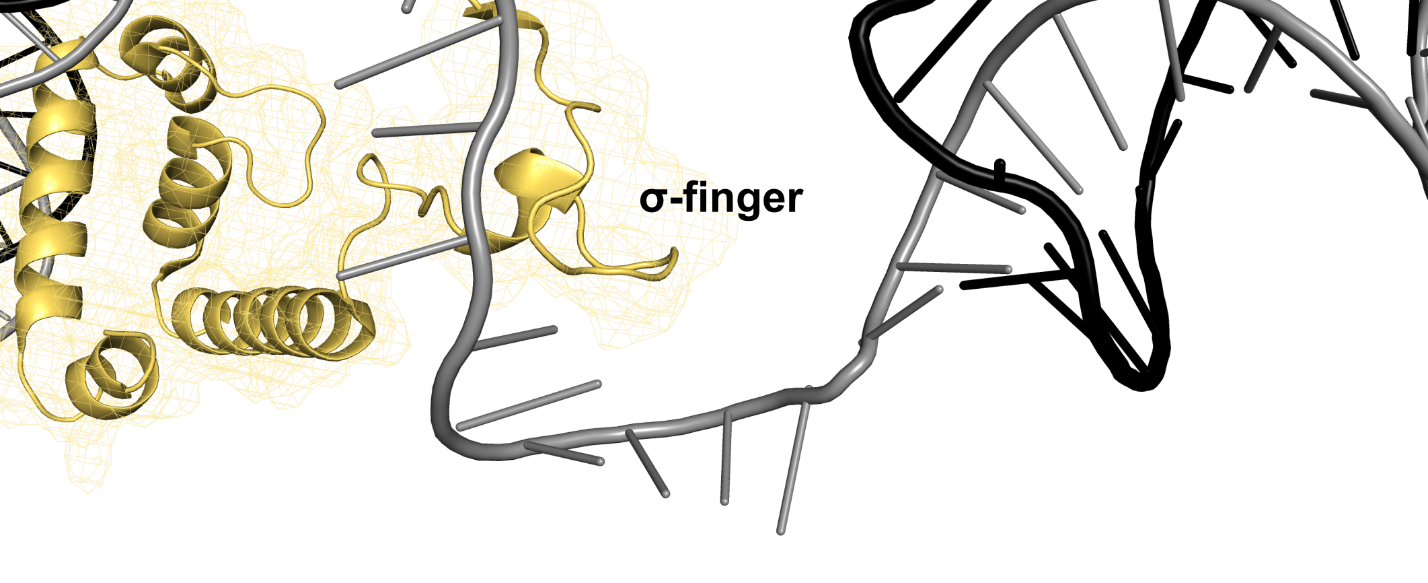


**Fig. S11.** Structure of the pR RP_o_ structure (7MKD). The σ^70^-factor is straw coloured; template DNA is in grey, and non-template DNA in black. The σ-finger is estimated to clash with a growing RNA chain of 4- to 6-nt in length.


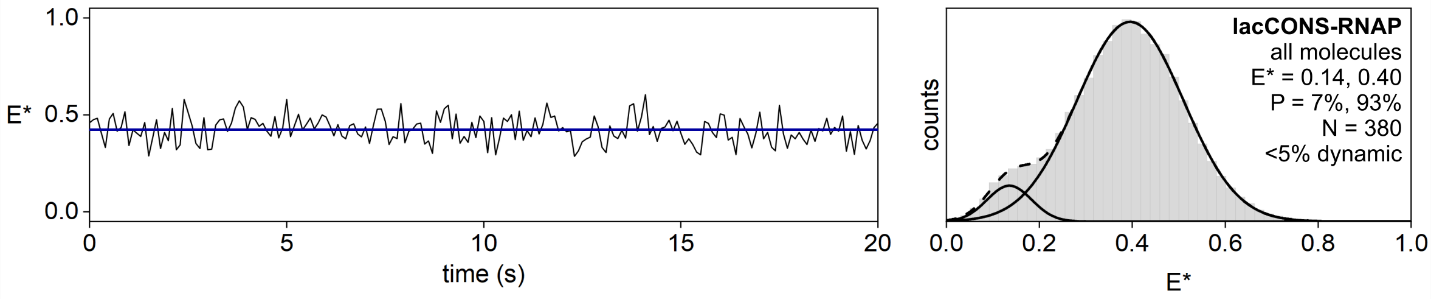


**Fig. S12:** smFRET data for the lacCONS DL RNAP-σ^70^ complex. Left, representative trace. Right, E* histogram with bi-modal Gaussian fitting (black line).

**
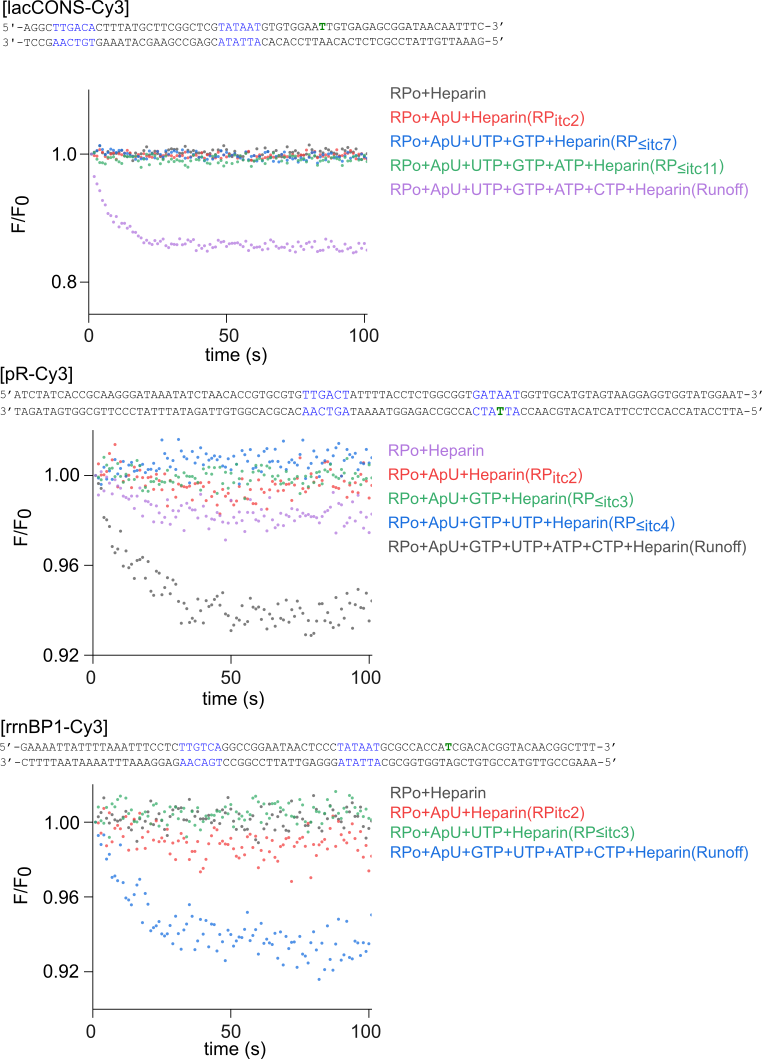
**

**Fig. S13**. **Promoter escape assay for A. pR and B. rrnBP1.** Sequences of pR-[-9-Cy3] and rrnBP1-[+2-Cy3] constructs used for the promoter escape assay are shown at the top of the panels. Fluorescence intensity decays corresponding to the addition of different subsets of NTPs at the bottom of the panels.


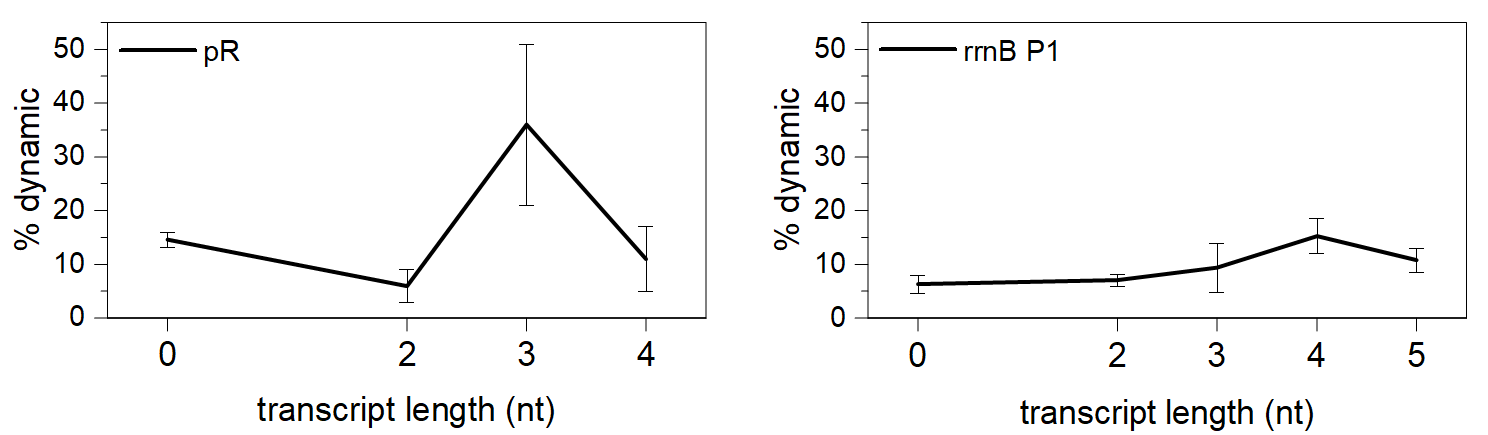
A

**Fig. S14:** Percentages of dynamic molecules observed for the experiments in Fig. 4 with the pR promoter and the rrnBP1 promoter.

**SI References**

1. Kalinin, S., Peulen, T., Sindbert, S., Rothwell, P.J., Berger, S., Restle, T., Goody, R.S., Gohlke, H., and Seidel, C.A.M. (2012). A toolkit and benchmark study for FRET-restrained high-precision structural modeling. Nat Methods *9*, 1218–1225. 10.1038/nmeth.2222.

2. Mukhopadhyay, J., Kapanidis, A.N., Mekler, V., Kortkhonjia, E., Ebright, Y.W., and Ebright, R.H. (2001). Translocation of σ70 with RNA Polymerase during Transcription: Fluorescence Resonance Energy Transfer Assay for Movement Relative to DNA. Cell *106*, 453–463. 10.1016/S0092-8674(01)00464-0.

3. Hudson, B.P., Quispe, J., Lara-González, S., Kim, Y., Berman, H.M., Arnold, E., Ebright, R.H., and Lawson, C.L. (2009). Three-dimensional EM structure of an intact activator-dependent transcription initiation complex. Proceedings of the National Academy of Sciences *106*, 19830–19835. 10.1073/pnas.0908782106.

4. Vrentas, C.E., Gaal, T., Ross, W., Ebright, R.H., and Gourse, R.L. (2005). Response of RNA polymerase to ppGpp: requirement for the ω subunit and relief of this requirement by DksA. Genes Dev. *19*, 2378–2387. 10.1101/gad.1340305.

5. Niu, W., Kim, Y., Tau, G., Heyduk, T., and Ebright, R.H. (1996). Transcription Activation at Class II CAP-Dependent Promoters: Two Interactions between CAP and RNA Polymerase. Cell *87*, 1123–1134. 10.1016/S0092-8674(00)81806-1.

6. Duchi, D., Bauer, D.L.V., Fernandez, L., Evans, G., Robb, N., Hwang, L.C., Gryte, K., Tomescu, A., Zawadzki, P., Morichaud, Z., et al. (2016). RNA Polymerase Pausing during Initial Transcription. Molecular Cell *63*, 939–950. 10.1016/j.molcel.2016.08.011.

7. Dulin, D., Bauer, D.L.V., Malinen, A.M., Bakermans, J.J.W., Kaller, M., Morichaud, Z., Petushkov, I., Depken, M., Brodolin, K., Kulbachinskiy, A., et al. (2018). Pausing controls branching between productive and non-productive pathways during initial transcription in bacteria. Nat Commun *9*, 1478. 10.1038/s41467-018-03902-9.

8. Holden, S.J., Uphoff, S., Hohlbein, J., Yadin, D., Le Reste, L., Britton, O.J., and Kapanidis, A.N. (2010). Defining the Limits of Single-Molecule FRET Resolution in TIRF Microscopy. Biophysical Journal *99*, 3102–3111. 10.1016/j.bpj.2010.09.005.

9. van de Meent, J.-W., Bronson, J.E., Wiggins, C.H., and Gonzalez, R.L. (2014). Empirical Bayes Methods Enable Advanced Population-Level Analyses of Single-Molecule FRET Experiments. Biophysical Journal *106*, 1327–1337. 10.1016/j.bpj.2013.12.055.

10. Duchi, D., Mazumder, A., Malinen, A.M., Ebright, R.H., and Kapanidis, A.N. (2018). The RNA polymerase clamp interconverts dynamically among three states and is stabilized in a partly closed state by ppGpp. Nucleic Acids Research *46*, 7284–7295. 10.1093/nar/gky482.
